# Affinity-matured B cell responses neutralizing type-I interferons underlie severe viral infections

**DOI:** 10.1016/j.cell.2026.04.013

**Published:** 2026-05-06

**Authors:** Morgane Fournier, Matthias Vanderkerken, Karim Dorgham, Paul Bastard, Olivia Ahouzi, Stephane Duquerroy, Ngoc Khanh Nguyen, Manon Broutin, Manon Charlet, Alexis Vandenberghe, Paolo Van Endert, Lucy Bizien, Omaira Da Mata-Jardin, Andrés Ferriño-Iriarte, Ahmed Haouz, Thibaut Belmondo, Sophie Hüe, Alessandro Borghesi, Carlos Rodríguez-Gallego, Donald C. Vinh, Evangelos Andreakos, Filomeen Haerynck, Rabih Halwani, Qiang Pan-Hammarström, Niklas K. Björkström, Benedikt Strunz, Trine H. Mogensen, COVID Human Genetic Effort, Antonio Piralla, Stefania Varchetta, Jorge Freixinet, Lucie Roussel, Sophie Trouillet Assant, Bénédicte Neven, Romain Levy, Tom le Voyer, Ottavia M. Delmonte, Cliona O'Farrelly, Jacques Rivière, Blanca Amador Borrero, Amélie Servettaz, Roger D. Kouyos, Daniel E. Kaufmann, Etienne Crickx, Marc Michel, Anne Puel, Laurent Abel, Charles-Edouard Luyt, Alexis Mathian, Kai Kisand, Darragh Duffy, Lluis Quintana-Murci, Zahir Amoura, Benjamin G. Hale, Jean-Claude Weill, Jean-Laurent Casanova, Felix A Rey, Guy Gorochov, Pascal Chappert, Matthieu Mahévas

**Affiliations:** 1. INEM, INSERM U1151/CNRS UMR 8253, Université Paris Cité, France.; 2. Sorbonne Université, INSERM, CIMI, Département d'Immunologie, AP-HP, Hôpital Pitié-Salpêtrière, Paris, France.; 3. Laboratory of Human Genetics of Infectious Diseases (LHGID), INSERM U1163, Necker Hospital for Sick Children, Paris, France.; 4. Imagine Institute, Université Paris Cité , Paris, France.; 5. LHGID, Rockefeller Branch, Rockefeller University, New York, USA.; 6. Department of Pediatric Immuno-Hematology and Rheumatology, CHU Necker-Enfants Malades, AP-HP, Paris, France.; 7. Institut Pasteur, Université Paris Cité, CNRS UMR 3569, Unité de Virologie Structurale, Paris, France.; 8. Université Paris-Saclay, Faculté des Sciences, Orsay, France.; 9. Service de Médecine Interne, CHU Henri-Mondor, AP-HP, UPEC, Créteil, France.; 10. Institut Pasteur, Université Paris Cité, CNRS UMR 3528, Crystallography Facility-C2RT, Paris, France.; 11. Département Immunologie-Hématologie, CHU Henri-Mondor, AP-HP, UPEC, Créteil, France.; 12. Host-Pathogen Interaction Group, San Matteo Research Hospital, Pavia, Italy.; 13. Department of Pediatrics, Gynecology and Obstetrics, University of Geneva, Geneva, Switzerland.; 14. Neonatal and Pediatric Intensive Care Unit, Mother, Child and Adolescent Department, Geneva University Hospitals, Switzerland.; 15. Department of Immunology, University Hospital Gran Canaria Dr Negrín, Canarian Health System, Las Palmas de Gran Canaria, Spain.; 16. Department of Medical and Surgical Sciences, School of Medicine, University of Las Palmas de Gran Canaria, and Instituto de Investigación Sanitaria de Canarias, Tenerife, Spain.; 17. Department of Clinical Sciences, University Fernando Pessoa Canarias, Las Palmas de Gran Canaria, Spain.; 18. CIBER de Enfermedades Respiratorias, Instituto de Salud Carlos III, Madrid, Spain.; 19. Department of Medicine, Division of Infectious Diseases, McGill University Health Centre, Montréal, and Infectious Disease Susceptibility Program, Research Institute-McGill, University Health Centre, Montréal, Canada.; 20. Laboratory of Immunobiology, Center for Clinical, Experimental Surgery and Translational Research, Biomedical Research Foundation of the Academy of Athens, Athens, Greece.; 21. Department of Pediatric Immunology and Pulmonology, CPIG, Jeffrey Modell Diagnosis and Research Centre, Ghent University Hospital, Ghent, Belgium.; 22. Department of Pediatrics and Internal Medicine, PID research laboratory, Ghent University, Ghent, Belgium.; 23. Research Institute for Medical and Health Sciences, College of Medicine, University of Sharjah, Sharjah, United Arab Emirates.; 24. Immunology Research Lab, College of Medicine, King Saud University, Riyadh, Saudi Arabia.; 25. Health and Wellbeing, NEOM, Tabuk, Saudi Arabia.; 26. Prince Fahad Bin Sultan chair for Biomedical Research, University of Tabuk, Tabuk, Saudi Arabia.; 27. College of Medicine, Alfaisal University, Riyadh, Saudi Arabia.; 28. Division of Immunology, Department of Biochemistry and Biophysics, Karolinska Institute, Stockholm, Sweden.; 29. Center for Infectious Medicine, Department of Medicine Huddinge, Karolinska Institute, Karolinska University Hospital, Stockholm, Sweden.; 30. Department of Infectious Diseases, Aarhus University Hospital, and Department of Biomedicine, Aarhus University, Aarhus, Denmark.; 31. Microbiology and Virology Department, Fondazione IRCCS Policlinico San Matteo, Pavia, Italy.; 32. Infectious Diseases Unit, Department of Internal Medicine, Fondazione IRCCS Ca' Granda Ospedale Maggiore Policlinico, Milan, Italy.; 33. Laboratory of Clinical Immunology, Division of Molecular Medicine, Rare Diseases and Advanced Therapy, Department of Experimental and Clinical Research, Fondazione IRCCS Policlinico San Matteo, Pavia, Italy.; 34. Department of Thoracic Surgery, University Hospital of Gran Canaria Dr. Negrín, Las Palmas de Gran Canaria, Spain.; 35. IUIBS, Las Palmas de Gran Canaria, Spain.; 36. Hospices Civils de Lyon, Lyon Sud Hospital, International Center of Research in Infectiology, Lyon University, INSERM U1111, CNRS UMR 5308, ENS, UCBL, Lyon, France.; 37. Laboratory of Clinical Immunology and Microbiology, Division of Intramural Research, NIAID, NIH, Bethesda, MD, USA.; 38. School of Medicine, Trinity Biomedical Sciences Institute, Trinity College, Dublin, Ireland.; 39. Pediatric Infectious Diseases and Immunodeficiencies Unit, Hospital Universitari Vall d'Hebron, Vall d'Hebron Barcelona Hospital Campus, Barcelona, Spain.; 40. Internal Medicine Department, Hôpital Lariboisière, AP-HP, Université Paris Cité, Paris, France.; 41. Service de médecine interne, faculté de médecine de Reims, CHU de Reims, and EA 7509, Université Reims Champagne Ardenne, Reims, France.; 42. Department of Infectious Diseases and Hospital Epidemiology, University Hospital Zurich, and Institute of Medical Virology, University of Zurich, Zurich, Switzerland.; 43. Division of Infectious Diseases, Department of Medicine, Lausanne University Hospital and University of Lausanne, Lausanne, Switzerland.; 44. Médecine Intensive Réanimation, Institut de Cardiologie, AP-HP, Pitié-Salpêtrière, Sorbonne-Université, and INSERM, UMRS_1166-ICAN, Paris, France.; 45. AP-HP, Pitié-Salpêtrière, Service de Médecine Interne 2, Institut E3M, INSERM, Paris, France.; 46. Molecular Pathology, Department of Biomedicine, Institute of Biomedicine and Translational Medicine, University of Tartu, Tartu, Estonia.; 47. Translational Immunology Unit, Institut Pasteur, Université Paris Cité, Paris, France.; 48. Human Evolutionary Genetics Unit, Institut Pasteur, Université Paris Cité, Paris, France.; 49. Chair Human Genomics and Evolution, Collège de France, Paris, France.; 50. Institute of Medical Virology, University of Zurich, Zurich, Switzerland.; 51. Howard Hughes Medical Institute, The Rockefeller University, New York, USA.; 52. Department of Pediatrics, Necker Hospital for Sick Children, Paris, France.; 53. INSERM U955, équipe 2, IMRB, UPEC, Créteil, France.; 54. Lead contact.

## Abstract

Autoantibodies neutralizing type-I interferons (AAN-I-IFNs) emerge as global, common, and strong determinants of a growing number of severe viral diseases. We report that AAN-I-IFNs^+^ patients with life-threatening COVID-19 pneumonia harbor circulating type-I IFNs-specific B cells indistinguishable from patients bearing T-cell tolerance defects of genetic origin. This autoimmune response mobilizes a highly diverse and stable circulating B-cell response that is detected prior to severe viral infection and acquires high affinity and neutralization potential to type-I IFNs through extended somatic hypermutation. X-ray crystallography and AlphaFold3 structural analysis of hundreds of patient-derived monoclonal antibodies reveals the extended breadth of this response, targeting three major B cell epitopes covering all facets of type-I IFNs. These findings support a model in which a germinal-center-derived memory B-cell response directed against type-I IFNs is established before severe viral infection, providing a core mechanism linking T-cell tolerance defect to pathogenic AAN-I-IFNs underlying severe viral diseases.

## Graphical Abstract

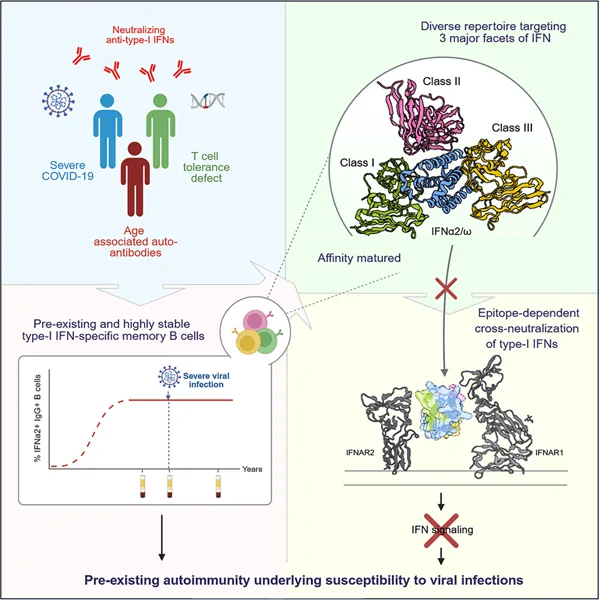

## Introduction

Human type-I interferons (IFNs) are essential for protective immunity to some viruses, as attested by rare monogenic inborn errors of the genes encoding type-I IFNs receptor or their response pathway.^1^ Autoantibodies (auto-Abs) neutralizing type-I IFNs (AAN-I-IFNs) are surprisingly frequent in the general population, as their prevalence rises sharply at the age of 65–70 years, from less than 0.4% to about 4%.^2,3^ AAN-I-IFNs impair the cell-protective antiviral effects of type-I IFNs by neutralizing the 12 subtypes of IFN-α, IFN-ω, and/or IFN-β.^4,5^ They underlie between 5 and 40% of cases of life-threatening COVID-19,^2,4,6^ seasonal influenza,^7^ MERS pneumonia,^8^ West Nile virus encephalitis,^9^ tick-borne virus encephalitis,^10^ as well as rarer arboviral diseases.^11^ They are likewise responsible for a third of fulminant viral hepatitis,^12^ as well as for rare case of lethal zoonotic H5N1 influenza.^13^ And, their pathogenic role has recently been extended to severe adverse reactions to live-attenuated vaccines in two studies in the context of the Yellow Fever^14^ and Chikungunya vaccines.^15^

The increase in AAN-I-IFNs prevalence in the elderly is unexplained. In younger individuals, AAN-I-IFNs may result from monogenic inborn errors of thymic epithelial cells (TECs) disrupting T-cell tolerance.^4^ This is classically seen in patients with autosomal recessive or dominant autoimmune polyendocrine syndrome type 1 (APS-1)^16–19^ due to deleterious germline variants of the autoimmune regulator (*AIRE*) gene,^20,21^ or with inborn errors of genes of the alternative NF-κB pathway affecting the development of AIRE-expressing medullary TECs.^22^ Patients with thymoma resulting in impaired AIRE expression also display AAN-I-IFNs.^23^ Similarly, AAN-I-IFNs may result from T cell-intrinsic defects, as illustrated by immunodysregulation polyendocrinopathy enteropathy X-linked syndrome caused by hemizygosity for deleterious germline variants of *FOXP3*,^24^ or combined immunodeficiency due to biallelic hypomorphic variants of the recombination-activating genes 1 or 2 (RAG1 or RAG2) (partial RAG deficiency, pRD)^25^. These auto-Abs are also observed in patients with conditions of less clear genetic architecture, such as myasthenia gravis (MG) and systemic lupus erythematosus (SLE).^26,27^ While MG is a thymic condition,^28^ SLE is more classically associated with impaired B-cell tolerance and the expansion of the T-bet-driven IgD^−^CD27^−^ unconventional autoreactive B-cell subsets (referred to as DN2) via the extrafollicular (EF) pathway.^29,30^

The evidence that AAN-I-IFNs are present before severe viral disease and could be causal for such disease in the general population is strong and has been reviewed elsewhere.^1,31^ Evidence is however indirect, as B cells specific for type-I IFNs have only been documented so far in APS-1 autoimmune patients.^19^ The increase in the prevalence of AAN-I-IFNs in the elderly population^2^ and the stepwise acquisition of reactivity to the various type-I IFNs^3^ are suggestive of a T-dependent B-cell response with progressive affinity maturation in germinal centers (GCs). Another, albeit less likely, possibility is that severe viral infection reveals an acquired defect of B-cell tolerance in older individuals, resulting in the extrafollicular expansion of unpurged autoreactive naive B cells, a phenomenon correlated with COVID-19 severity.^32^ These questions are biologically relevant — as AAN-I-IFNs may be seen as a first robust marker of thymic tolerance deficits^33^ — and medically important, due to their frequency in the general population and the growing range of life-threatening viral infections shown to be associated with them.^34^ We approached these questions by leveraging access to samples collected in a tightly defined temporal window post-hospitalization in life-threatening COVID-19 patients during the first two waves of the SARS-CoV-2 pandemic, compared to patients with a known T cell etiology for AAN-I-IFNs, to perform a detailed single-cell and structural analysis of the underlying initial B cell response. Evidence of stability, affinity maturation before SARS-CoV-2 infection and highly similar responses in both cohorts, points towards a common underlying T cell tolerance breakdown in all patients.

## Results

### Analysis of patients with AAN-I-IFNs, with or without life-threatening COVID-19

To investigate the cellular basis of type-I IFNs-specific antibody responses in patients with severe viral infections — including the potential for an acute breach of self-tolerance and the recruitment of *de novo* autoreactive naive B cells through the extrafollicular pathway — we retrospectively analyzed blood samples from 29 patients with AAN-I-IFNs, life-threatening COVID-19 (S-CoV) and available sampling in the very early phase of SARS-CoV-2 infection (i.e. < 15 days after hospitalization) obtained through the international COVID Human Genetic Effort consortium (Figure 1A, S1A, Table S1 and STAR Methods). As controls, we collected samples at similar time points from S-CoV patients without AAN-I-IFNs (*n*=11) and samples from post-pandemic healthy donors (HD, *n*=14). Finally, we included samples from a second cohort of 20 patients with known, genetic and/or autoimmune etiologies of AAN-I-IFNs, referred to here as “immune tolerance defect” (ITD) patients. Part of these patients had inborn errors of immunity (IEI; NF-κB2 deficiency (*n*=4), APS-1 (*n*=6), pRD (*n*=2)) but this cohort also included patients with thymoma (*n*=2), thymoma with Good’s syndrome immunodeficiency (GS, *n*=3) or systemic lupus (SLE, *n*=3) (Figure 1A and Table S1).

### High-affinity and cross-reactive IFN-α2/IFN-ω-specific IgG^+^ B cells in patients with life-threatening COVID-19 and AAN-I-IFNs

Most studies have focused on the detection of circulating neutralizing Abs against type-I IFNs. For single-cell analysis of the underlying IFN-α2/IFN-ω-specific B-cell response, we first optimized a multiparametric flow cytometry panel including fluorescent tetramers for IFN-α2 and IFN-ω, to allow the simultaneous phenotypic, V(D)J repertoire, functional and structural analysis of IFN-α2 and IFN-ω (IFN-α2/ω)-specific B cells (Figure 1A and S1B-E). Analysis of all samples from the HD, S-CoV and ITD cohorts revealed the presence of a detectable IFN-α2 tetramer^+^ IgG^+^ B-cell population in 26 of 29 S-CoV patients with AAN-I-IFNs and in 16 of 20 ITD patients (Figure 1B-C), but in none of the control AAN-I-IFNs^−^ S-CoV (n=11) and post-pandemic healthy controls (*n*=14). IFN-α2 tetramer^+^ IgG^+^ B cells were rare — often with only a few tens of cells per vial — particularly in some ITD donors with low peripheral B-cell counts. However, IFN-α2 tetramer^+^ IgG^+^ B cells were even detectable in four of six S-CoV or ITD patients with low AAN-I-IFN titers and in all subgroups of ITD donors (Figures S1F), but only in three of five GS/thymoma patients and one of the three SLE patients analyzed. Only a few IFN-ω tetramer-only IgG^+^ B cells could be detected in any given donor (Figure S1E and data not shown).

In both AAN-I-IFNs^+^ S-CoV and ITD patients, most monoclonal antibodies (mAbs) derived from IFN-α2/ω tetramer^+^ IgG^+^ B cells bound IFN-α2 (Figure 1D) or IFN-ω (Figure 1E) with mid to high affinity (88.9% and 85.5% of S-CoV and ITD clones respectively), displaying KDs below 10^−9^ M for most. Just under half of the IFN-α2-binding clones cross-reacted with IFN-ω (52.2% and 40.5% of S-CoV and ITD clones, respectively), with similar affinities for both IFN-ω and IFN-α2 (Figure 1E). Similar results were obtained in type-I IFN neutralization assays, with 51.8% and 25.9% of the tested monoclonal anti-IFN-α2-specific antibodies efficiently neutralizing IFN-α2 or IFN-ω, respectively, at a concentration of 0.1 ng/ml (Figure 1F). Cross-reactivity to IFN-β was less frequent, with less than a quarter of all the mAbs tested having a detectable, but lower, affinity (13.6% and 6.2% of S-CoV and ITD clones, respectively, Table S2B) and none displaying detectable IFN-β neutralization *in vitro* (Figure 1F). Similar populations of high-affinity IFN-α2-specific IgG^+^ circulating B cells displaying potent cross-neutralization of IFN-α2 and IFN-ω are thus readily detectable at an early stage of SARS-CoV-2 infection in AAN-I-IFNs^+^ S-CoV patients and in ITD patients.

### Highly mutated and diverse repertoire of IFN-α2/ω specific IgG^+^ B cells in AAN-I-IFN^+^ patients with life-threatening COVID-19 or immune tolerance defects

The *de novo* expansion of a T-bet-driven IgD^−^CD27^−^CD21^low^CD11c^+^ DN2 subset, displaying few VDJ mutations and specificity for autoantigens, has recently been reported in life-threatening COVID-19.^32^ An analysis of our own cohort confirmed such a strong increase in the proportion of DN2 cells among circulating B cells in AAN-I-IFNs^+^ S-CoV patients (Figure S1D), but interestingly not in most AAN-I-IFNs^−^ S-CoV and ITD patients. By contrast, expansion of the antibody-secreting cell (ASC) population could be observed in all S-CoV patients, regardless of the presence of AAN-I-IFNs (Figure S1C). To investigate a potential causal link between the overactivated EF response and AAN-I-IFNs in S-CoV patients, we next performed single-cell VDJ sequencing on all previously characterized as well as on additionally sorted IFN-α2 and IFN-ω-specific IgG^+^ B cells. In total, we obtained 183 antibody heavy-chain (IgH) sequences and 134 paired light-chain (IgL) sequences from 11 S-CoV individuals at an early time point (<2 weeks post hospitalization, in median 7 days after hospital admission [0-14], all from patients sampled during the first or second waves of the COVID-19 epidemic), and 328 IgH sequences from 13 ITD patients (163 paired IgL sequences). IFN-α2/ω-specific clones from early S-CoV patients displayed high mutation loads in both IgH and IgL V genes (V_H_ and V_L_) (median of 21 mutations per V_H_, mean of 21.67±0.67), with very few unmutated sequences (Figure 2A, Figure S2A and Table S2). We observed the same trend in all patients analyzed (Figure S2B-C), including those sampled on the first day of COVID-19 hospitalization (see S-CoV60, Figure S2C). ITD patients displayed slightly higher V_H_ and V_L_ mutational loads (median of 24 mutations per V_H_, mean of 24.46±0.89, *P*=0.0171, Figure 2A, Figure S2A and Table S2), but this appeared to be mostly driven by APS-1 patients (Figure S2B-C).

V_H_ gene usage of IFN-α2/ω-specific IgG^+^ B-cell clones from S-CoV (<2wks post hospitalization) and ITD patients displayed a common bias towards IGHV1–8 and IGHV1–69 (Figure 2B), in preferential association with IGKV1–33 or IGKV1–39 and IGKV1–33 or IGKV4–1, respectively (Figure S2D). We also observed a modest overrepresentation of IGHV1–3 (paired with IGKV3–20), IGHV3–9 and IGHV3–43 in S-CoV patients relative to published IgG memory B cell (MBC) reference repertoires,^35^ although we cannot exclude donor-driven bias for these less represented V_H_ genes. In both patient cohorts, irrespective of etiology, the IFN-α2/ω-specific IgG repertoires showed signs of clonal expansion but retained similar diversity (Figure 2C and Figure S2E-F). We also did not observe any major differences in CDR3 characteristics, including length, hydrophobicity, net charge and polarity (Figure S2G), with only a minor increase in CDR3 length in IFN-α2/ω-specific IgG^+^ B-cell clones from ITD patients.

IFN-α2-specific DN2 cells were detectable in half of S-CoV patients but only accounted for a small proportion of total IFN-α2-specific IgG^+^ B cells (Figure 2D). Instead, IFN-α2-specific IgG^+^ B cells were found mostly in the activated B-cell (ABCs) (CD27^+^ CD21^low^CD71^+^) and resting memory B-cell (rMBCs) (CD27^+^CD21^+^CD71^−^) pools (Figure 2D), with similar numbers of V_H_ mutation in all IFN-α2-specific IgG^+^ B-cell subsets (Figure 2E). These findings thus indicate a minor, if any, recruitment of autoreactive naive IFN-α2–specific B cells in parallel to the ongoing anti-viral response in S-CoV patients. Instead, IFN-α2–specific B cells seen in both S-CoV and ITD patients appear to share characteristics of affinity-matured MBCs usually observed after 3–6 months of germinal-center maturation in the context of infection or vaccination.^36–39^

### IFN-α2/ω–specific memory B cells pre-exist SARS-CoV-2 infection

To confirm the pre-existence of IFN-α2/ω–specific MBCs in the context of severe SARS-CoV-2 infection, we next assessed their presence in rare PBMC samples of documented AAN-I-IFNs^+^ patients from the pre-COVID-19 era. One of the included APS-1 patients from our ITD cohort had a documented severe COVID-19 in March 2020 with sampling before (2012) and after (2023). Analysis of IFN-α2/ω–specific IgG^+^ B cells from both samples did not reveal any major change in clonal diversity or mutational load over the decade and the severe infection that occurred in between sampling time points (Figure 2F-H). To extend this observation to individuals with no known autoimmune etiology, we next analyzed IFN-α2/ω–specific B cells in pre- or post-pandemic samples from 8 individuals carrying age-associated anti–type-I IFNs neutralizing autoantibodies (AAb) (Figure 2I, Table S1 and STAR Methods). This notably included pre-mid2019 samples from three individuals in the Swiss HIV Cohort Study who later developed severe COVID-19.^3^ IFN-α2–specific B cells were readily detectable in 6 out 8 of these individuals (3 out of 4 of each sub-cohorts), at frequencies comparable to those observed in AAN-I-IFNs^+^ severe COVID-19 and ITD (Figure 2J and 2K). Most importantly, IFN-α2/ω-specific B cells from the pre-mid 2019 samples already harbored numbers of somatic mutations in their V_H_ gene usually associated with several months of GC maturation in human^36–39^ (Figure 2L and Table S2), in line with our initial observation in recently hospitalized S-CoV patients. Overall, these results strongly support the notion of IFN-α2/ω–specific MBCs’ pre-existence prior to severe SARS-CoV-2 infection.

### High-affinity anti-IFN-α2 auto-Abs display hallmarks of germinal-center-driven affinity maturation

To confirm that observed V(D)J somatic mutations indeed reflected affinity maturation within GCs, we next expressed and validated 34 representative mAbs from eight S-CoV patients, including three mAbs monospecific for IFN-ω (STAR Methods and Table S3). Eleven amongst the fully characterized high-affinity anti-IFN-α2 Abs were further selected for expression of their unmutated common ancestor (UCA), representing B-cell receptors isolated from seven different S-CoV donors, bearing a diverse set of V_H_/V_L_ pairings and targeting the three main epitopes being recognized on IFN-α2 (see below and Figure 5A). In line with the presence of mutated residues in the complementarity-determining regions (CDRs) of these antibodies (Table S3), all germline-reverted UCA displayed a strong drop in affinity towards IFN-α2 (median of 112.4-fold decrease, range of 12 to over 10^3^) (Figures 3A). This was associated with a parallel affinity drop towards IFN-ω for cross-reactive UCA (Figure 3B). In most cases, the gain acquired by somatic hypermutation (SHM) corresponded to a shift from micromolar to nanomolar equilibrium dissociation constants, essentially driven by lower dissociation rates (K_off_) (Figure 3C). And, probably due to the faster dissociation rates, none of the germline-reverted Abs was able to neutralize IFN-α2 signaling (Figure 3D). These results thus confirmed affinity maturation as a key step in the development of high-affinity neutralizing anti-type-I IFNs pathogenic antibodies in humans.

The fact that 9 of the 11 reverted Abs retained detectable affinity (KD < 10^−7^) for IFN-α2 (n=8) or IFN-ω (n=1) (Figure 3A-B) also suggested the presence of germline B-cell receptors with pre-existing reactivity to type-I IFNs in the naive B-cell repertoire. To distinguish low affinity germline reactivity to type-I IFNs from broad auto/polyreactivity, we further assessed the levels of auto/polyreactivity of our re-expressed IFN-α2-specific mAbs and their respective UCA. Amongst the 32 tested native recombinant antibodies, about 30% displayed polyreactivity against LPS, insulin, and DNA, and 50% were autoreactive, as demonstrated by positive Hep-2 reactivity at mid-high mAb concentration (Figure S3A-B). However, a similar analysis on paired native/UCA mAbs revealed that these anti-IFN-α2 autoreactive/polyreactive properties were mostly gained through SHM in the GC (Figures 3E-F and S3C-F), except for one mAb (GMAB03), whose UCA displayed lower but still detectable polyreactivity (Figure S3C-F).

### IFN-α2/ω -specific IgG^+^ B cells represent a stable memory population post-infection

Our observations on the APS-1 patient sampled more than one decade apart, also indicated that once formed, the anti-IFN-α2/ω autoimmune B cell response is very stable and long lasting. To extend this observation to S-CoV patients, we next analyzed samples from AAN-I-IFNs^+^ S-COV patients collected after recovery (> 3 months and up to 3 years post-hospitalization (*n*=14, Figure 4A, Table S1)), including longitudinal samples from four patients. This confirmed the remarkable stability of the IFN-α2/ω-specific B-cell response over time in terms of cell frequencies (Figure 4B and 4C), mutational load (101 IgH and 34 IgL sequences from 7 S-CoV patients, Figure 4D, Figure S2H), repertoire (Figure 4E), affinity and neutralization potency for recombinant IFN-α2 or IFN-ω (Figure 4F-G and Table S3B), and phenotype (Figure 4H). Additional rounds of GC maturation, however, cannot be excluded as seen in one of the three patients with longitudinal early and late post-COVID-19 V(D)J sequencing data, with some clones displaying continued acquisition of V_H_ mutations overtime (Figure S2I-J).

Altogether, these findings point to IFN-α2/ω-specific IgG^+^ B cells as a germinal-center derived long-lasting memory repertoire that both predate and outlast severe infection and most likely stem from the recruitment of naive B cells bearing intrinsic low germline reactivity to IFN-α2 or IFN-ω. The similar patterns of affinity, V_H_ somatic mutations and diversity of the anti-IFNα2/ω humoral response of individual AAN-I-IFNs^+^ S-CoV and ITD patients further suggest a common broad breakdown of T-cell tolerance associated with the generation of a diverse set of high-affinity, highly mutated anti-IFN-α2/ω MBC clones.

### AAN-I-IFNs from life threatening COVID-19 patients target three major antigenic sites on IFN-α2

To gain further insight into the structural basis of IFN-α/ω recognition by auto-Abs in S-CoV patients, we made use of the 34 validated, expressed, IFN-α2 and/or IFN-ω-specific mAbs from S-COV patients described above, adding a fourth IFN-ω monospecific mAb isolated from the dominant splenic clone of an AAN-I-IFNs^+^ AAb organ donor (Table S2 and Table S3). A competitive binding assay performed with these mAbs together with previously published anti-IFN-α2 human or humanized mAbs (rontalizumab^40^ and sifalimumab^41^, respectively) revealed the existence of three distinct epitope groups (Figures 5A-B and S4A). One group of mAbs competed with the high-affinity IFNAR2 subunit (Group I) and contained all the IGHV1–8 clones, together with rontalizumab. A second group competed with IFNAR1 and, to a lesser extent, IFNAR2 (Group II), whereas the third group, containing sifalimumab, competed solely with IFNAR1 (Group III) (Figure 5A-B). Antibodies displaying IFN-α2/IFN-ω cross-reactivity belonged almost exclusively to Group I (Figure 5A, 5C and 5E). Similar experiments with IFN-ω showed one IFN-ω-specific antibody (GMAB07, Group I (IFN-ω only)) in competition with other Group I cross-reactive antibodies and three IFN-ω-monoreactive antibodies clustered in a separate epitope group (Group “other” (IFN-ω only), Figure 5C). These groups of antibodies were present in all donors (Figure 5D) and IFN-α2-neutralizing auto-Abs were equally distributed between groups (Figure 5E), with intragroup differences in neutralization potency largely explained by higher dissociation rates for non-neutralizing mAbs (Figure S4B).

### Crystal structures of AAN-I-IFN complexes with type-I IFNs

We further improved the delineation of the targeted epitopes in these three groups by using X-ray crystallography. We obtained three different crystals of IFN-α2 in complex with one, two or three of the non-competing antibodies with IFN-α2 (Figure 5F-H), diffracting to a resolution of 2.0, 1.8 and 4.0Å, respectively (Figure S4C). The resulting X-ray structures (Figure 5F-H) showed that the epitopes of the group I antibodies overlap with the IFNAR2 binding site, with that of PMAB15 spanning the short helix in the A-B loop and helix E of IFN-α2, as previously described for rontalizumab (PDB 4Z5R)^40^, whereas PMAB03 also contacts helix A. The epitope of PMAB14 (Group II) mostly involves helix D, on a surface facing away from the membrane side in the IFNAR2/IFN-α2/IFNAR1 sandwich complex on cells. This mAb partially clashes with the IFNAR1 N-terminal fibronectin type III domain (termed SD1) in the signaling complex. The binding of PMAB14 to IFN-α2 had a small but detectable impact on both the position of the A-B loop of IFN-α2 and the binding of PMAB15 as part of ternary complex 2. Finally, the epitope of PMAB19 (Group III) lies on the opposite side of IFN-α2 to that of Group I mAbs, encompassing residues on helices B and C, as previously described for sifalimumab (PDB 4YPG)^41^ and competing with the SD2 and SD3 domains of IFNAR1.^42^ Together, these data reveal the contact sites of the members of all three groups identified in our epitope-binning experiments (Figure 5I) and confirmed the presence of mutated V_H_/V_L_ residues in the paratope of these antibodies (Figure S4D-E).

### AI-based predictions reveal common and structurally diverse recognition of IFN-α2 in COVID-19 and ITD patients

To extend the above observations to all sequenced mAbs in our dataset, including mAbs from ITD patients, we made use of the recent release of the artificial intelligence (AI) model AlphaFold 3.^43^ Structural interaction with IFN-α2 was predicted with confidence (“confident”, *n*=54) to very high confidence (“very high confidence”, *n*=113) for 167 of the 317 analyzed IFN-α2-specific IgH/IgL pairs (Figure S5A, Table S2C and STAR Methods). We had available X-ray crystallography and epitope-binning data for 3 and 23 of these 167 mAbs, respectively. The predicted structures were essentially identical to the experimental X-ray structures described above (Figure 6A-B, S5B-C and Tables S4-7), apart from the highly flexible loop around Gln 46 of IFN-α2. We observed perfect agreement between the three groups identified by epitope-binning experiments and the AlphaFold3-predicted epitope groups (Figure S6). AlphaFold3 predicted most structures of IFN-α2/mAb complexes to belong to the same three main classes characterized by the X-ray structures (Figures 6C-F and Figure S6), with comparable frequencies, including overrepresentation of Class I mAbs, in all cohorts (Figure 6E). Unsupervised clustering of all 167 predictions further allowed us to split each main class into two to three distinct sub-classes based on more subtle positioning of the heavy and/or light chain on IFN-α2 (Figure 6F). These variations included additional binding on either sides of helix A of IFN-α2, as predicted for GMAB03 (Class IIIb) or members of Class Ic (Figure 6F and Figure S6). They also helped explain cross-group competitions observed in our epitope-binning assay, as nicely exemplified by Class IIa PMAB02 mAb whose orientation and positioning closer to the end of helix D explain the partial competition seen against some Class I mAbs (Figure 5A, Figure 6F and Figure S6). Four out of the 167 mAbs were further predicted to bind to the front loops connecting all main helices of IFN-α2 (“Front” class, Figure 6F and Figure S6). These predictions remain to be experimentally validated by X-ray crystallography.

Overall, these structural data highlight the diversity of targeted epitopes over the entire surface of IFN-α2 (Figure 6C-E) down to the individual donors’ level (Figure 5D and 6F). The exact frequencies, however, should be considered with caution, as the accuracy of AlphaFold3 predictions appears affected to some degree by IGHV usage (e.g. IGHV3–23), targeted epitopes (e.g. Class II) and CDR3 length (Figure S5D, S5F and S5G).

### IFNs-specific auto-Abs cross-reactive or polyreactive potential is epitope-dependent

A key observation brought by serum analysis regarding the development of AAN-I-IFNs has been the description of progressive acquisition of neutralization from one to all type-I IFNs.^3^ To investigate this, we performed additional AlphaFold3-based predictions using IFN-ω as the antigen, leading to 44 “confident” to “very high confidence” predictions out of 75 identified IFN-ω reactive IgH/IgL pairs in our dataset (Figure 7A and Figure S5A and S5E-G). Cross-analyzing these predictions in the light of ex vivo affinity and neutralization data (Figure 1D-F, Figure 5E and Table S2) showed that IFN-α2/IFN-ω cross-neutralizing antibodies exclusively belonged to Classes Ia and Ib (Figure 7A), which dominate both IFN-ω and IFN-α2-specific responses (Figure 6E and Figure S7A). Anti-IFN-ω Abs belonging to classes II and III were predicted, and the later notably included two of the three re-expressed IFN-ω mono-specific mAbs identified as belonging to the “other (IFN-ω only)” epitope-binning group. But these two classes exhibited only limited cross-reactivity potential (Figure 7A).

Investigating cross-reactivity to other IFN-α subtypes and reanalyzing our poly- and auto-reactivity data considering our structural modeling showed an overall oppositive picture. Broad reactivity towards all IFN-α subtypes was mostly observed for Class II and III mAbs, whereas a large part of Class I mAbs lacked total reactivity to all or part of IFN-α1/4/5/7/8/10/16/17 and 21 (Figure 7B). Similarly, polyreactivity and autoreactivity traits in IFN-α2-specific antibodies appeared mostly restricted to Class II and/or Class III mAbs, with a strong enrichment in polyreactive mAbs among Class III (Figure 7C). This is in line with the strong conservation of helix D residues between all IFN-α subtypes but not IFN-ω and the amino-acid differences at the end of the helix A and the beginning of the A-B loop observed between IFN-α subtypes (Figure S7B). An overall positively charged pocket serving as a key contact site for Class I mAbs is notably progressively lost in IFN-α subtypes poorly recognized by IFN-α2-specific mAbs but not on IFN-ω (Figure 7D-E). In contrast, the Class II and mostly Class III epitope displayed patches of negative charges (Figure 7D and Figure S7C-D), largely lost on IFN-ω. This was associated with mostly positively charged HCDR3 amongst Class III mAbs, with reduced frequencies of acidic residues (Figure 7F), two features previously associated with polyreactive antibodies.^44–47^ Shorter HCDR3 lengths, reduced polarity and slightly increased hydrophobicity were also present (Figure S7E), although the latter two traits were shared with other sub-classes of anti-IFN-α2 mAbs (Figure S7F). These properties appeared further lost on antibodies targeting the IFN-ω version of the Class III epitope (Figure S7G), which included two of the non-polyreactive Class III mAbs.

Affinity-matured polyreactive B cells have been described in HIV and influenza, where polyreactivity is thought to promote naive B cell selection,^48^ affinity selection^49^ or antibody plasticity^49^ and broad reactivity.^50^ Our results, coupled with our germline reversion experiments (Figure 3), mostly suggest that epitope specific constraints instruct the recruitment and affinity maturation of a diverse repertoire of autoantibodies. On one side, broad reactivity is limited by differences amongst structural properties of type-I IFNs. On the other side, polyreactivity acquisition is likely a consequence of optimized binding towards the Class III epitope of IFN-α subtypes.

## Discussion

Direct evidence for the presence of AAN-I-IFNs before life-threatening viral disease has been limited to rare patients for whom blood samples drawn before infection could be retrospectively retrieved and tested.^3,9,16^ The best evidence so far that these auto-Abs precede severe viral disease comes from the Swiss longitudinal cohort of HIV-infected patients; once these auto-Abs appear, they persist and gain in neutralization capacity and cross-reactivity to other type-I IFNs over a few years to decades.^3^ Yet, only a handful of human type-I IFNs-specific mAbs have been described to date,^19,40,41^ limiting the conclusions that can be drawn about their natural history. Our findings unambiguously indicate that type-I IFN-specific B cells in patients with life-threatening COVID-19 represent a diverse GC-derived memory B cell population with long-lasting potential and whose establishment predate SARS-CoV-2 infection. This is likely to be the case also for the growing number of severe viral diseases reported to be associated with these auto-Abs.^7,9–11^

The list of monogenic etiologies underlying AAN-I-IFNs in individuals under 65 years of age (~0.4%) is expanding, yet the basis for the ~4% prevalence in the elderly remains unclear. The high somatic hypermutation and affinity maturation of IFN-α2-specific MBC clones are hallmarks of GC responses,^51^ which have been characterized in detail in only a few autoimmune diseases. In contrast to cryoglobulinemic vasculitis, where tolerance escape has been proposed to arise from somatic mutations outside IGVH in a single clone,^52^ the broad clonal diversity of type-I IFN-specific B cells in COVID-19 patients mirrors that seen in ITD patients with broad T-cell tolerance defect. Moreover, targeting of multiple epitopes across IFN-α2 and IFN-ω argues against molecular mimicry, unlike EBV-driven cross-reactivity reported in multiple sclerosis,^53^ where unmutated ancestors lack detectable self-reactivity.

Together with similarities between patients with life-threatening COVID-19 and immune tolerance defects, these findings support GC engagement of IFN-reactive naive B cells present in the normal repertoire, driven by autoreactive T cells that escape central or peripheral tolerance. Age-related thymic decline may promote the emergence of such T cells,^54^ potentially explaining the surge in AAN-I-IFNs around age 70, analogous to tolerance failures in thymoma.^23^ Germline-reverted anti-IFN-γ antibodies with IFN binding,^55^ and the frequent IFN-α2 reactivity of germline-reverted antibodies here, indicate that type-I IFN-reactive naive B cells are common but require GC maturation to become pathogenic. This is consistent with historical reports that therapeutic IFN-α or IFN-β can induce AAN-I-IFNs.^56,57,58^ Recurrent endogenous type-I IFN production during infections or environmental exposures may further promote AAN-I-IFNs in individuals with defective T cell tolerance. Overall, AAN-I-IFNs likely reflect a spectrum of etiologies shaped by genetic predisposition, aging, and environmental triggers.^54^

Another open question is whether the progressive emergence of neutralizing antibodies to multiple type-I IFNs^3^ reflects progressive broadening of cross-reactivity by a few clones or stepwise recruitment of additional clones targeting new epitopes. To date, only a few human anti–IFNs antibodies have been described (from APS-1 patients^19^ or scFv libraries^59^), and none neutralize all type-I IFNs despite evidence of sustained affinity maturation in patients with APS-1. Our functional, epitope-binning, and X-ray data on an expanded antibody set indicate that cross-reactivity to IFN-α2 and IFN-ω is largely restricted to antibodies targeting an epitope overlapping the IFNAR2-binding site (Class I), which undergo parallel affinity maturation to both cytokines but show limited breadth across IFN-α subtypes, as reported for rontalizumab.^19^ AlphaFold3-based predictions extended these observations, defining three major classes and up to 7 sub-classes, of IFN-α2/ω-binding antibodies with distinct HCDR3 constraints and opposing properties regarding the ability to cross-react to IFN-α2 and IFN-ω or to target all IFN-α subtypes. Together, these data support progressive recruitment and affinity maturation of naive B cells targeting distinct epitopes, to explain the drift from one to multiple targeted type-I IFNs over time. This is consistent with studies in mice showing that once tolerance is broken for one self-antigen, autoreactive GCs can generate B cells targeting other self-antigens in a mechanism known as epitope spreading.^61^

Overall, our findings show that type-I IFN-specific B cells in AAN-I-IFNs^+^ patients constitute a diverse, GC-derived memory B-cell compartment with long-lived potential, whose establishment predates severe SARS-CoV-2 infection and most likely other severe viral infections. While this strongly suggests a breakdown of T cell tolerance to type-I IFNs as a core mechanism across patients, the question of a parallel breakdown in B cell tolerance as well as the contributions of genetic and environmental determinants of AAN-I-IFNs remain to be determined, especially in the elderly population. The unique toolbox and atlas of type-I IFN-targeting human antibodies reported in this study also provides a solid basis for the design of new approaches targeting such autoantibodies. The identification of type-I IFNs–specific B cells paves the way for their selective depletion, while the mapping of B-cell epitopes on type-I IFNs provides a basis for the development of decoy molecules that would bind autoantibodies without engaging type-I IFNs receptors.^62^

### Limitations

All AAN-I-IFNs^+^ S-CoV and ITD patients included in this study had detectable neutralizing autoantibodies to both IFN-α2 and IFN-ω. Similar studies in individuals with autoantibodies neutralizing only one of these molecules would be needed to confirm our predictions regarding the early stages of this autoimmune B-cell response. Because we primarily focused on the IgG^+^ response in S-CoV patients, we also cannot exclude the possibility of a parallel low-affinity IgM extrafollicular response, as has been reported for other self-antigens.^32^ Additionally, we were limited in our VDJ repertoire analysis at the single donor level by the relatively limited number of donors in which we could sequence significant number of IFN-α2/ω-specific B cells. This reflects the inherently low frequency of IFN-α2/ω-specific and total circulating B cells in severe COVID-19 patients^32^ and most patients with primary immunodeficiencies.^22,29^

### RESOURCE AVAILABILITY

#### Lead Contact

Further information and requests for resources and reagents should be directed to and will be fulfilled by the Lead Contact, Matthieu Mahévas (matthieu.mahevas@aphp.fr).

#### Materials Availability

Materials generated in this study will be made available upon reasonable request and may require a material transfer agreement.

#### Data and Code Availability

Coordinates and structure factors of the crystal structures have been deposited in the Protein DataBank (PDB) and have been made publicly available. Accession numbers are listed in the key resources table. Single cell culture VDJ sequencing data reported in Figure 2, 4 and Figure S2 are included in Table S2. All sequences used for small scale monoclonal antibodies expressions and all data generated using these antibodies are included in Table S3. Dedicated wrapper functions used for repertoire analysis and interaction with AlphaFold3 models are available here: https://github.com/PChappert/RERB. Any additional information required to reanalyze the data reported in this paper is available from the lead contact upon request.

#### Consortia

The members of COVID Human Genetic Effort are Alessandro Aiuti, Saleh Al-Muhsen, Alexandre Belot, Catherine M. Biggs, Alexandre Bolze, Anastasiia Bondarenko, Ahmed A. Bousfiha, Petter Brodin, John Christodoulou, Aurélie Cobat, Roger Colobran, Antonio Condino-Neto, Stefan N. Constantinescu, Munis Dündar, Sotirija Duvlis, Sara Espinosa-Padilla, Jacques Fellay, Carlos Flores, José Luis Franco, Filomeen Haerynck, David Hagin, Lennart Hammarström, Elena W. Y. Hsieh, Yuval Itan, Emmanuelle Jouanguy, Elżbieta Kaja, Kai Kisand, Davood Mansouri, Isabelle Meyts, Giuseppe Novelli, Satoshi Okada, Maria Papadaki, Rebeca Perez de Diego, Jordi Perez-Tur, David S. Perlin, Jonny Peter, Garyphallia Poulakou, Carolina Prando, Aurora Pujol, Laurent Renia, Carlos Rodríguez-Gallego, Nikoletta Rovina, Mohammad Shahrooei, Pere Soler-Palacín, Vassili Soumelis, Şehime Gülsün Temel, Stuart E. Turvey, Mohammed J. Uddin, Mayana Zatz, Qian Zhang, Shen-Ying Zhang, Jean-Laurent Casanova.

The members of the Swiss HIV Cohort Study are Abela IA, Aebi-Popp K, Anagnostopoulos A, Bernasconi E, Braun DL, Bucher HC, Calmy A, Cavassini M (Chairman of the Clinical and Laboratory Committee), Ciuffi A, Dollenmaier G, Egger M, Elzi L, Fehr JS, Fellay J, Frigerio Malossa S, Fux CA, Günthard HF, Hachfeld A, Haerry DHU (deputy of “Positive Council”), Hasse B, Hirsch HH, Hoffmann M, Hösli I, Huber M, Jackson-Perry D (patient representatives), Kahlert CR, Kaufmann D, Keiser O, Klimkait T, Kouyos RD, Kovari H, Kusejko K (Head of Data Centre), Labhardt ND, Leuzinger K, Martinez de Tejada B, Marzolini C, Metzner KJ, Müller N, Nemeth J, Nicca D, Notter J, Paioni P (Chairman of the Mother & Child Substudy), Pantaleo G, Perreau M, Rauch A (President of the SHCS), Salazar-Vizcaya LP, Schmid P, Segeral O, Speck RF, Stöckle M, Surial B, Tarr PE, Trkola A, Wandeler G (Chairman of the Scientific Board), Weisser M, Yerly S.

## STAR METHODS

### EXPERIMENTAL MODEL AND STUDY PARTICIPANT DETAILS

#### Study participants

We enrolled 29 patients (24M/5F) with proven hypoxemic COVID-19 pneumonia (life-threatening (WHO scale score≥5),^63^ ranging from severe to critical, including 27 patients in intensive care units) and documented AAN-I-IFNs. Patients were recruited from seven countries (Belgium, Canada, France, Greece, Italy, Sweden, United Arab Emirates, Sweden) through the international COVID Human Genetic Effort consortium (www.covidhge.com) (Table S1). Twenty-five of the 29 included patients from that cohort were infected during the first or second waves of the COVID-19 pandemic, between March 2020 and early 2021. All had available frozen PBMC samples from within 15 days of hospitalization, with most (two-thirds) having been sampled during the first week (Table S1). Plasma at the time of sampling from all these patients contained Abs able to neutralize IFN-α2 and IFN-ω at a concentration of 0.1 ng/ml and most (26/29 patients) contained Abs able to neutralize IFN-α2 at a concentration of 10 ng/ml (Figure S1A). The neutralization of IFN-β, however, was limited to a minority of patients (8 of 29). Additional PBMCs could be collected at a later time point after infection for four of these 29 patients, between three months and 3 years after infection, to analyze the stability of the anti-type I IFN B cell response (Table S1). Another ten patients with documented AAN-I-IFNs, but no early sampling post hospitalization, were further recruited after recovery from COVID-19, with PBMCs collected between three months and three years post hospitalization (Table S1). As controls, we also enrolled 11 patients with life-threatening COVID-19 without AAN-I-IFNs from whom samples had been collected at a similar early time point post-infection (<15 days of hospitalization) and who were also infected during the first or second waves of the COVID-19 pandemic, between March 2020 and early 2021 (Table S1 and^64^). All individuals were recruited according to protocols approved by local Institutional Review Boards (IRBs): Policlinico San Matteo (Pavia, Italy, IRB20200037602), Research Institute of the McGill University Health Care (Montréal, Québec, Canada, 10–256GEN), Dubai Scientific Research Ethics Committee (Dubai, United Arab Emirates, DSREC-08/2021_14), Karolinska Institute (Stockholm, Sweden, Drn 2020–01558), Ghent University Hospital (Gent, Belgium, code BC07574), ‘Sotiria’ General Chest Diseases Hospital (Athens, Greece, approval numbers 16707/10–7-18 and 8385/31–3-20) and Ile-de-France VI ethics committee for Henri-Mondor University Hospital (Assistance Publique-Hôpitaux de Paris, France, MEMO-COV-2 study (NCT04402892), approval number 40–20 HPS). Finally, we also included 14 post-pandemic healthy donors (included in the aforementioned MEMO-COV-2 study), 20 patients with AAN-I-IFNs associated with immune tolerance defects of known etiology (approved by Ile-de-France II and CERAPHP center ethics committees, IRB 2010-A00634–35 and IRB 00011928, respectively) and 8 individuals with age-associated autoantibodies, including pre-mid2019 samples from three individuals in the Swiss HIV Cohort Study (SHCS), who later developed severe COVID-19,^3^ and from one individual included in the French “Milieu Intérieur” cohort,^6^ as well as samples collected during the autumn of 2021 from four individuals identified as AAN-I-IFNs^+^ AAb, with no documented history of severe SARS-CoV-2 infection (Table S1). Written informed consent was obtained from all participants. For all patients, plasma samples were used in immunoassays testing for the presence of neutralizing auto-Abs against type-I IFNs. All donor-related informations are listed in Table S1.

### METHOD DETAILS

#### Interferon neutralization assay

The *in vitro* blocking activity of anti–IFN-α2 and anti–IFN-ɷ auto-Abs – polyclonal patient plasma, single-IgG^+^ B-cell culture supernatants or re-expressed monoclonal antibodies - was determined in a reporter luciferase activity. Briefly, HEK293T cells were transfected with a plasmid containing the firefly luciferase gene under the control of the human ISRE (Interferon-Stimulated Response Element) promoter in the pGL4.45 backbone and a plasmid constitutively expressing the *Renilla* luciferase for normalization (pRL-SV40). Cells were transfected in the presence of the X-tremeGENE9 transfection reagent (Sigma-Aldrich, reference number 6365779001) for 24 hours. Cells in Dulbecco’s modified Eagle medium (DMEM; Thermo Fisher Scientific) supplemented with 2% fetal calf serum and 10% healthy donor or patient plasma (after inactivation at 56°C for 20 min) were either left unstimulated or were stimulated with IFN-α2a (Miltenyi Biotec, reference number 130–108-984) or IFN-ω (Merck, reference number SRP3061) at a concentration of 10 ng/ml or 0.1 ng/ml or with IFN-β (Miltenyi Biotec, reference number 130–107-888) at 1 ng/ml for 16 hours at 37°C. Each sample was tested once for each cytokine and dose. Finally, cells were lysed for 20 min at room temperature, and luciferase levels were measured with the Dual-Luciferase Reporter 1000 Assay System (Promega, reference number E1980) according to the manufacturer’s protocol. Luminescence intensity was measured with a VICTOR-X Multilabel Plate Reader (PerkinElmer Life Sciences, USA). Firefly luciferase activity values were normalized against *Renilla* luciferase activity values. These values were then normalized against the median induction level for non-neutralizing samples and expressed as a percentage (“Residual IFN signaling (% of max)” in figures). Plasmas or monoclonal antibodies (whether re-expressed or supernatants from single-cell cultured IFN-α2/ω ^+^ IgG^+^ B cells) were considered neutralizing if luciferase induction, normalized against *Renilla* luciferase activity, was below 15% of the median value for controls tested the same day (dotted red line on figure panels).

#### Protein biotinylation and tetramer preparation

Recombinant human IFN-α2a (rhIFN-α2a, Miltenyi Biotec, reference number 130–108-984), recombinant human IFN-ω (rhIFN-ω, Merck, reference number SRP3061) and bovine serum albumin (BSA) proteins were biotinylated with EZ link NHS biotin (Thermo Fisher Scientific) according to the manufacturers’ instructions. Tetramers were produced immediately before staining, by incubating the biotinylated proteins with fluorochrome-conjugated streptavidin at a molar ratio of 4:1 for 1 hour at 4°C (PE/BV421, BV711/BV421 and PECy5-conjugated streptavidins for IFN-α2a, IFN-ω and decoy BSA tetramers, respectively). We then added 2.4 ng free biotin and incubated for 10 minutes before mixing the tetramers.

#### Flow cytometry and cell sorting

Peripheral blood mononuclear cells (PBMCs) were isolated from blood samples via standard density-gradient centrifugation and stored at −150°C. Cells were slowly thawed in RPMI-1640 (Gibco) supplemented with 10% fetal bovine serum (FBS, Gibco), washed twice and incubated with a mixture of rhIFN-α2 and rhIFN-ϖ tetramers (equivalent to 200 ng of each protein) and fluorochrome-conjugated antibodies (1:200 for CD14, 1:100 for CD19, CD38, CD3, and IgD, 1:50 for CD21, CD11c, CD27 and IgG) in a final volume of 100 μl phosphate-buffered saline (PBS, Gibco)-2% FBS for 40 min on ice. Cells displaying nonspecific binding were excluded by adding a non-relevant tetramer constructed with biotinylated bovine serum albumin to the mixture. A mouse anti-human interferon α/β receptor chain 2 monoclonal antibody (anti-IFNAR2, Merck, reference number MAB1155, 1:100) was also added to the mixture to limit undesirable binding of the rhIFN-α2a/rhIFN-ω tetramers to the IFN-α/β receptor. Viable cells were identified with a LIVE/DEAD Fixable Aqua Dead Cell Stain Kit (Thermo Fisher Scientific, 1:30) and incubated with conjugated antibodies. Samples were acquired on ARIA II SORP (BD Biosciences) or SONY MA900 cell sorters and IFN-α2- and IFN-ω-specific IgG^+^ B cells were sorted in 96-well plates in ultra-purity mode. E*x vivo* culture of single-cell sorted IFN-α2 tetramer^+^ IgG^+^ B cells (see below) confirmed an overall purity of this staining above 90% with our final panel (Panel 3, Figure S1E), which was then used for all cell sorting that did not include an initial *in vitro* culture validation step. In this panel, IFN-α2/IFN-ω-specific IgG^+^ B cells were identified within the CD19^+^IgG^+^IgD^−^ B-cell population using a combination of four tetramers (IFN-α2 PE, IFN-α2 BV421, IFN-ω BV421 and IFN-ω BV711), that included the use of a common color (BV421) for both proteins (see Figure S1B and S1E). Some limited staining could also be seen in naive and non-naive IgM^+^ B cells, albeit only around 30% of them showed IFN-α2 or IFN-ω reactivity by ELISA in initial in vitro single-culture validation studies and none by BLI or neutralization assay (data not shown). We also did not detect any clonal relationship between IFN-α2 tetramer^+^ IgG^+^ memory B cells and naive and non-naive IgM^+^ B cells identified as part of our tetramer staining (data not shown), raising questions regarding the contribution of these cells to the pathogenic anti-type-I IFNs response in these patients. Data were analyzed with dedicated SONY and FlowJO software. Detailed gating strategies for cell sorting and analysis are depicted in Figure S1.

#### Single-cell culture

Single-cell cultures were performed as previously described^65^. Single IFN-α2 and IFN-ϖ tetramer^+^ B cells were sorted in 96-well plates containing MS40L^lo^ cells expressing CD40L (kindly provided by G. Kelsoe, ^66^). Cells were cultured at 37°C, under an atmosphere containing 5% CO_2_ for up to 25 days, in RPMI-1640 (Invitrogen) supplemented with 10% HyClone FBS (Thermo Scientific), 55 μM 2-mercaptoethanol, 10 mM HEPES, 1 mM sodium pyruvate, 100 units/ml penicillin, 100 μg/ml streptomycin, and minimum essential medium non-essential amino acids (all from Invitrogen), supplemented with recombinant human BAFF (10 ng/ml), IL2 (50 ng/ml), IL4 (10 ng/ml), and IL21 (10 ng/ml; all from Peprotech). Part of the supernatant was carefully removed on days 4, 8, 12, 15 and 18 and the same amount of fresh medium supplemented with cytokines was added to the cultures. On days 21 and 25, the supernatants of single-cell cultures were harvested, frozen, and stored at −20°C. At the end of the culture period, the cell pellets were placed on ice and gently washed with PBS (Gibco) before being resuspended in 50 μl RLT buffer (Qiagen) supplemented with 1% β-mercaptoethanol. They were then frozen and stored at −80°C until further processing.

#### ELISA

Total IgG and rhIFN-α2- and rhIFN-ω-specific IgG from culture supernatants were determined in a custom-developed ELISA. We coated 96-well ELISA plates (Thermo Fisher Scientific) with goat anti-human Ig (10 μg/ml, Invitrogen) in 0.1M sodium carbonate, pH 9.6, for 1 h at 37°C or with rhIFN-α2a (Miltenyi Biotec, 130–108-984) and rhIFN-ω proteins (Peprotech, 3000–2J), each at 5 μg/ml, overnight at 4°C. After blocking, cell culture supernatants were added and incubated with the plates for 1 h. The reaction was then developed with HRP-conjugated goat anti-human IgG (1 μg/ml, Immunotech) and tetramethylbenzidine substrate (TMB, Eurobio). OD_450_ and OD_620_ were measured, and supernatants with OD_450_-OD_620_ (OD_450–620_) values above 0.09 were considered positive for IFN-α2 or IFN-ω.

In a similar manner, autoreactivity and polyreactivity of re-expressed monoclonal antibodies were determined using a commercial kit and a custom-developed ELISA, respectively. We used an anti-nuclear antibody (ANA) ELISA kit (Werfen, 066708750) according to the manufacturer’s instructions, and we coated 96-well ELISA plates (Thermo Fisher Scientific) with double-stranded DNA (Merck, D4522), LPS (Merck, L2880), insulin (Merck, I9278), at 20, 10 and 5 μg/ml respectively, overnight at 4°C. After blocking, a four-fold titration of each antibody (with concentrations starting at 4 μg/ml, then 1, 0.25 and 0.065 μg/ml) was added and incubated with the plates for 90 minutes. The reaction was then developed with HRP-conjugated goat anti-human IgG (1 μg/ml, Immunotech) and tetramethylbenzidine substrate (TMB, Eurobio). OD_450_ and OD_620_ were measured, and antibodies with OD_450_-OD_620_ (OD_450-_620) values above 0.09 for HEP-2 nuclear antigens or for all the 3 antigens tested (dsDNA/LPS/insulin) were considered autoreactive and polyreactive, respectively. In some figures (Figures 3E-F and 7C), number of re-expressed monoclonal antibodies tested is specified at the middle of the donut plot.

#### IgH sequencing and monoclonal antibody re-expression

At the end of single-cell culture, the B-cell pellets were resuspended in 50 μl RLT Buffer (Qiagen), frozen and stored at −80 °C. Successfully cultured clones (IgG concentration ≥ 1 μg/ml and positive results for rhIFN-α2 and rhIFN-ϖ-specific ELISA at day 21–25) were selected and extracted with the NucleoSpin96 RNA extraction kit (Macherey-Nagel) according to the manufacturer’s instructions. Reverse transcription was then performed with the SuperScript IV enzyme (Thermo Fisher Scientific) in a final volume of 14 μl (42°C 10 min, 25°C 10 min, 50°C 60 min, 94°C 5 min) with 4 μl RNA and random hexamers (Thermo Fisher Scientific). Polymerase chain reaction (PCR) was then performed as described by Tiller et al^46^. Briefly, 3.5 μl cDNA was used as a template and amplification was performed in a total volume of 40 μl of a mixture containing forward L-VH, L-Vλ or L-Vκ primers and reverse Cγ, Cλ or Cκ primers for heavy chains, λ and κ light chains, respectively, with the HotStar^®^ Taq DNA polymerase (Qiagen) and 50 cycles of PCR (94°C 30 s, 58°C 30 s, 72°C 60 s). PCR products were sequenced with the reverse primer CHG-D1 and read on an ABI PRISM 3130XL genetic analyser (Applied Biosystems). Sequence quality was verified with CodonCode Aligner software (CodonCode Corporation) or directly within R using the sangeranalyseR R package v1.14.0.

5’ PCR1 Primer Mix:

VH :

*5′ L-VH 1 ACAGGTGCCCACTCCCAGGTGCAG

*5′ L-VH 3 AAGGTGTCCAGTGTGARGTGCAG

*5′ L-VH 4/6 CCCAGATGGGTCCTGTCCCAGGTGCAG

*5′ L-VH 5 CAAGGAGTCTGTTCCGAGGTGCAG

*5’L-VH 2 CCTTCATGGGTCTTGTCCCAGATCACC

*5’ L-VH 6 CCATGGGGTGTCCTGTCACAGGTACAG

*5’ L-VH 7 GCAACAGGTGCCCACTCCCAGGTGCAG

Vκ :

*5′ L Vκ 1/2 ATGAGGSTCCCYGCTCAGCTGCTGG

*5′ L Vκ 3 CTCTTCCTCCTGCTACTCTGGCTCCCAG

*5′ L Vκ 4 ATTTCTCTGTTGCTCTGGATCTCTG

Vλ :

*5′ L Vλ 1 GGTCCTGGGCCCAGTCTGTGCTG

*5′ L Vλ 2 GGTCCTGGGCCCAGTCTGCCCTG

*5′ L Vλ 3 GCTCTGTGACCTCCTATGAGCTG

*5′ L Vλ 4/5 GGTCTCTCTCSCAGCYTGTGCTG

*5′ L Vλ 6 GTTCTTGGGCCAATTTTATGCTG

*5′ L Vλ 7 GGTCCAATTCYCAGGCTGTGGTG

*5′ L Vλ 8 GAGTGGATTCTCAGACTGTGGTG

3’ PCR1 Primers:

VH :

*3′ Cγ (IgG) CH1 GGAAGGTGTGCACGCCGCTGGTC

Vκ :

*3′ Cκ 543 GTTTCTCGTAGTCTGCTTTGCTCA

Vλ :

*3′ Cλ CACCAGTGTGGCCTTGTTGGCTTG

Additionally, for some patients and time points, some paired IgH and IgL sequences were obtained directly by single cell sorting in 4 μl lysis buffer containing PBS (Gibco), DTT (Thermo Fisher Scientific) and RNAsin (Promega). Reverse transcription and a first PCR were performed as described above (50 cycles), followed by a second 50-cycle PCR with 5’AgeI VH, Vλ or Vκ primer mix and reverse Cγ, Cλ or Cκ primers for heavy chains, λ and κ light chains, respectively, before sequencing.

5’ PCR2 Primer Mix :

VH :

*5’AgeI VH1/5 CTGCAACCGGTGTACATTCCGAGGTGCAGCTGGTGCAG

*5′ AgeI VH3 CTGCAACCGGTGTACATTCTGAGGTGCAGCTGGTGGAG

*5′ AgeI VH4 CTGCAACCGGTGTACATTCCCAGGTGCAGCTGCAGGAG

*5’ AgeI VH 6–1 CTGCAACCGGTGTACATTCCCAGGTACAGCTGCAGCAG

*5’ AgeI VH2 CTGCAACCGGTGTACATTCCCAGATCACCTTGAAGGAG

*5’ AgeI VH7 CTGCAACCGGTGTACATTCCCAGGTGCAGCTGGTCCAA

Vκ :

*5′ Pan Vκ ATGACCCAGWCTCCABYCWCCCTG

Vλ :

*5′ AgeI Vλ 1 CTGCTACCGGTTCCTGGGCCCAGTCTGTGCTGACKCAG

*5′ AgeI Vλ 2 CTGCTACCGGTTCCTGGGCCCAGTCTGCCCTGACTCAG

*5′ AgeI Vλ 3 CTGCTACCGGTTCTGTGACCTCCTATGAGCTGACWCAG

*5′ AgeI Vλ 4/5 CTGCTACCGGTTCTCTCTCSCAGCYTGTGCTGACTCA

*5′ AgeI Vλ 6 CTGCTACCGGTTCTTGGGCCAATTTTATGCTGACTCAG

*5′ AgeI Vλ 7/8 CTGCTACCGGTTCCAATTCYCAGRCTGTGGTGACYCAG

3’ PCR2 primers :

VH :

*3’ D1 (IgG) TTCGGGGAAGTAGTCCTTG

Vκ :

*3′ Cκ 494 GTGCTGTCCTTGCTGTCCTGCT

Vλ :

*3′ XhoI Cλ CTCCTCACTCGAGGGYGGGAACAGAGTG

Six monoclonal antibodies were first re-expressed in house as recombinant proteins after the subcloning of VH and VL genes into pFUSE vectors (InvivoGen) containing the human IgG1 constant region and the human kappa/lambda constant region, as reported elsewhere ^67^. HEK293T cells were cotransfected with the expression vectors in a 1:1 ratio, with the JetPrime transfection reagent (Polyplus-Transfection) and after 6 days of culture, the supernatants were analyzed for antibody expression by ELISA. The antibodies present were purified on a protein A affinity column connected to an AKTA chromatography system (GE Healthcare) and concentrated with ultrafiltration membranes with a molecular weight cutoff of 30 kDa.

An additional 29 monoclonal antibodies, and the unmutated common ancestor (germline reverted) form from 11 of our 35 re-expressed antibodies, were re-expressed by Proteogenix SAS. Briefly, the cDNAs encoding the variable regions of the heavy and light chains were chemically synthesized, with optimization for expression in CHO cells, and subcloned in ProteoGenix’s proprietary mammalian cell expression vectors containing backbones for the human IgG1 heavy chain constant region and the human kappa/lambda light chain constant region. An endotoxin-free DNA preparation method was used for the construction of each vector and the vectors were then used to transfect XtenCHO cells with the XtenCHO transfection protocol, in a total volume of 3.5 ml. The culture medium was collected 8 days after transfection and purified by one-step affinity purification (Protein A). Final quality control was performed by spectrophotometric measurements of A_280nm_ and qualitative and quantitative analyses by SDS-PAGE.

In total, type-I IFN-specific B cells could be sequenced from 11 severe COVID-19 patients, ranging across close to five decades (median 62 years old [21–78]) (see Table S2B). Antibodies were re-expressed from 8 of these severe COVID-19 patients (median 55.5 years old [21–78]) and one donor with age-associated AAN-I-IFNs, with 7 and 4 of our reverted antibodies coming from donors having ≤35 and ≥59 years old respectively (see Table S3A) and 8 from sequences obtained in the first 3 days post-hospitalization.

#### Computational analyses of VDJ sequences

Processed FASTA sequences from the sequencing of heavy and light chains from sorted single cells were annotated with Igblast v1.19.0 against the human ImMunoGeneTics (IMGT) reference database. Clonal cluster assignment (DefineClones.py) was performed with the Immcantation/Change-O toolkit^68^ on all heavy-chain V sequences. Sequences that had the same VH-gene, same JH-gene, including ambiguous assignments, and the same HCDR3 length with a maximal length-normalized nucleotide hamming distance of 0.15 were considered likely members of the same clonal group. Clonal group were further corrected based on information from available light chains using the resolveLightChains() function from the Dowser v2.3 R package^69^, prior to germline reconstruction using the createGermline() function from the same package. Mutation frequencies in V genes, V_H_ distributions, HCDR3 properties and repertoire diversities statistics were then calculated using the calcObservedMutations() and countGenes() functions from the SHazaM v1.2.0 R Packages and aminoAcidProperties() and alphaDiversity() functions from the Alakazam v1.3.0 R package respectively. Public datasets for naive and IgG^+^ cells from healthy donors were downloaded from the cAb-Rep database^35^ and included donors from Vander Heiden, J. A. et al.^70^, Rubelt, F. et al.^71^, Galson, J. et al. ^72^and Briney,B. et al^73^. Clonal representations were generated with the circlize v0.4.16 R package. Donut charts represent the clonal distribution of B cells from indicated samples/donors, with slices size corresponding to, and arranged according to, clones’ sizes. Total number of sequences is indicated in the middle of each donut chart. In some (Figure 2C), slices are colored according to clone rank. In other (Figures 2G and 4E), colored slices indicate persistent clones isolated at both time points and grey slice correspond to non-persisting expanded clones. Singlets, non-persisting, are always grouped in the remaining white area. The outer black semi-circle indicates the proportion of sequences belonging to expanded clones (two or more clonally related sequences), with frequencies further indicated on the top right of the donut plot in Figure 2C. Lineage trees were inferred and plotted using Dowser v2.3 R package.

All analyses were performed with R v.4.4.1 on a macOS Sonoma 14.4.1 (Apple M1 Pro) system. Numbers of included sequences are indicated at the bottom (Figures 2A, 2H and 4D), top (Figure 2E and 2L) or middle (Figure 2C, 2G and 4E) of each graph.

#### Affinity measurement by biolayer interferometry

Affinity for various type-IFNs was assessed by biolayer interferometry assays on an Octet Red96 instrument (Sartorius). Anti-Human Fc Capture (AHC) biosensors (18–5060) were immersed in supernatants from single-cell cultures of B cells or in solutions of re-expressed monoclonal antibodies (5 μg/ml) at 25°C for 500 seconds. Biosensors were equilibrated by incubation for 5 minutes in 1x PBS buffer with 0.1% BSA and 0.01% Tween 20 surfactant (PBS-BT) before measurement. Association was allowed to occur for 240 s in PBS-BT with individual type-I IFNs at a concentration of 100 nM, followed by dissociation for 480 s in PBS-BT. Between cycles, biosensors were regenerated by three rounds of alternating cycles of regeneration buffer (10 mM glycine HCl, pH 2.0) for 30 s and PBS-BT for 30 s. We subtracted the reference sensor (unloaded sensor) and reference well (additional association cycle in the absence of ligand) traces from the curves and full curve fitting was performed with a local 1:1 binding model in HT Data analysis software 11.1 (ForteBio). Four mAbs with IFN-α2a or IFN-ω association curves between 0.05 and 0.1 nm were further retested with a concentration of 300 nM of each cytokine for validation of the measured KD. The following type-I IFNs were used: rhIFN-α2a (Merck, H6041, expressed in HEK293 cells, Figure 1D-E); rhIFN-α2a (Miltenyi Biotec, 130–108-984, expressed in *E.coli*, Figures 5A, 5E, S4A-B); rhIFN-ω (Merck, SRP3061, expressed in *E.coli*, Figures 1E, 5C, 5E); rhIFN-α1/2a/4/5/6/7/8/10/14/16/17/21 (Bio-Techne (11014-IF; 10984-IF; 10998-IF; 11017-IF; 11168-IF; 11079-IF; 11018-IF; 11016-IF; 11019-IF; 11156-IF; 11082-IF and 11159-IF, respectively, all expressed in HEK293 cells, IFN-β (Peprotech, Peprotech (300–02BC)), expressed in CHO cells, Figure 7B).

Epitope-binning assays were further performed on IFN-α2 and IFN-ω for 34 re-expressed IFN-α2/ω-specific monoclonal antibodies (mAbs) isolated from AAN-I-IFN^+^ S-CoV as well as two previously published anti- IFN-α2 antibodies (rontalizumab^40^ and sifalimumab^41^), using a classical epitope-binning sandwich assay approach (Figure 5B). For each antibody tested in epitope-binning experiments, anti-human Fc capture (AHC) biosensors (18–5060) were first immersed in a solution of the re-expressed monoclonal antibody (5 μg/ml) or in a solution of published anti-IFNα2 monoclonal antibodies (rontalizumab, sifalimumab, 5 μg/ml) at 25°C for 600 seconds. Biosensors were then quenched by incubating loaded biosensors in polyclonal human IgG (10 μg/ml) solution for 300 s. Association with rhIFN-α2a (Miltenyi Biotec, 130–108-984) or rhIFN-ω (Sigma, SRP3061) was allowed to occur for a further 300 s in PBS-BT at 100 nM (or at 300 nM for association with rhIFNAR1-Fc or with lower-affinity monoclonal Abs). Competitive binding was then assessed with a predefined set of re-expressed monoclonal antibodies representing the major epitope groups identified at a concentration of 5 μg/ml or with rhIFNAR1-Fc and rhIFNAR2-Fc (Sinobiological, 13222-H02H and 10359-H02H, respectively) at a concentration of 10 μg/ml in PBS-BT for 300 s. Between cycles, biosensors were regenerated by three rounds of alternating cycles of regeneration buffer (10 mM glycine HCl, pH 2.0) for 30 s and PBS-BT for 30 seconds.

Heatmap visualization of epitope-binning results and measurements of affinity for all IFNs-α and IFN-ω were generated with the ComplexHeatmap v2.20.0 R package. For affinity against all IFNs-α and IFN-ω, log10-transformed KD values were used and plotted monoclonal antibodies were grouped by epitope-binning groups, with additional unsupervised row and column clustering within groups.

#### Protein production and purification

##### Protein production

Codon-optimized synthetic DNA sequences encoding the variable domains of PMAB03, PMAB14, PMAB15, and PMAB19 were inserted into a modified pMT/BiP (Invitrogen) vector, designated pT350, for production as single-chain variable fragments (scFvs) in the *Drosophila* S2 expression system. All sequences included the *Drosophila* BiP signal peptide at the N-terminus (MKLCILLAVVAFVGLSLG), and a double-strep tag

(AGWSHPQFEKGGGSGGGSGGGSWSHPQFEK) preceded by an enterokinase cleavage site (DDDDK) at the C-terminus. The interferon alpha 2 (IFN-α2) sequence was cloned for expression in S2 cells. The construct generated for this purpose contained a thrombin cleavage (LVPRGS) site followed by a histidine tag (HHHHHHHH) at the C-terminus.

Plasmids (2 μg) were used to transfect *Drosophila melanogaster* Schneider line 2 cells (S2) in the presence of the Effectene^®^ transfection reagent (Qiagen), according to the manufacturer’s instructions. The cells were cotransfected with the pCOPuro plasmid at a 1:20 ratio for puromycin selection. After 24 h, stable cell lines were selected by adding 8 μg/ml puromycin (Invivogen) to the culture medium. The transfected cells were maintained and amplified at 27°C in Hyclone™ SFM4Insect™ medium with L-glutamine (Cytiva), supplemented with 1% penicillin/streptomycin antibiotic cocktail (Gibco). Protein expression was induced by adding 4 μM CdCl_2_ to 1 L of cell culture for each scFv and 3 L for IFN-α2. After 4–5 days of induction, the stably transfected S2 cell supernatants were harvested and concentrated to approximately 50 ml with the Vivaflow^®^ 200 ultrafiltration system with a 10 kDa cutoff (Sartorius). The scFv supernatants were then supplemented with 0.24% BioLock Biotin blocking solution (IBA) and 0.1 M Tris-HCl pH 8.0 and centrifuged for 30 minutes at 20,000 × *g*.

##### Purification

For the scFvs, the process began with affinity purification on StrepTactin HP resin (Cytiva) based on binding of the double-strep tag. The antibodies were initially eluted in a buffer containing 10 mM Tris-HCl pH 8.0, 150 mM NaCl, 1 mM EDTA, and 2.5 mM desthiobiotin (IBA). The eluate was subjected to buffer exchange with 20 mM Tris/HCl + 150 mM NaCl pH8.0 (SEC buffer) supplemented with 10 mM Tris/HCl pH 8.0 + 10 mM CaCl_2_ to facilitate proteolytic removal of the C-terminal tag enterokinase (NEB) used at a ratio of 1:1000 (v/v).

After 16 hours of incubation at room temperature, a second StrepTactin affinity-column purification was performed to confirm tag removal, with the protein no longer binding to the column and instead being collected earlier in the wash buffer containing 10 mM Tris-HCl, 150 mM NaCl, and 1 mM EDTA at pH 8.0. PMAB14 and PMAB19 scFv samples were then concentrated and supplemented with deglycosylation buffer, containing 0.1 M NaOH and 0.1 M NaCl at pH 5.7, together with endoglycosidase-D and endoglycosidase-H (NEB) at a ratio of 1:2500 (v/v). This deglycosylation reaction was performed overnight at room temperature. To halt the digestion and deglycosylation reactions effectively, 1% PMSF (v/v) was added.

Affinity purification of the recombinant IFN-α2 was achieved with a HisTrapTM HP column (Cytiva), with elution in 20 mM Tris (pH 8.0), 200 mM NaCl, and 500 mM imidazole. No cleavage was performed to remove the histidine tag from this sample.

After affinity purification, all proteins (scFvs and IFN-α2) were subjected to size exclusion chromatography to remove potential aggregates. We used a Superdex 200 column (Cytiva) equilibrated in SEC buffer for this purpose. The scFvs and IFN-α2 were then mixed together to produce an IFN-α2+PMAB15 complex (complex 1) at a 2:1 ratio, an IFN-α2+PMAB15+PMAB14 complex (complex 2) at a 1:1:1 ratio and an IFN-α2+PMAB03+PMAB14+PMAB19 complex (complex 3) at a 3:1:1:1 ratio. These complexes were concentrated to 2.5 mg/ml, 27.1 mg/ml, and 4.7 mg/ml, respectively, with the Vivaspin Centricon system for crystallization trials.

#### Crystallization and structure determinations

For the high-throughput crystallization screen, we used the vapor diffusion method with a MosquitoTM nanodispensing system (STPLabtech, Melbourn, UK) according to the established protocols of the Crystallogenesis facility of Institut Pasteur.^74^ Manual optimization was performed in Linbro plates with the hanging-drop method by mixing 2 μl protein sample with 4 μl reservoir solution. Crystals of complex 1 (PMAB15 (Group I)/IFN-α2b) grew in 0.2 M ((NH_4_)_2_SO_4_, 0.1 M sodium acetate (pH 4.6), supplemented with 30% PEG MME 2000, with diffraction to a resolution of 2.0 Å resolution. Crystals of complex 2 (PMAB15 (Group I)/PMAB14 (Group II)/IFN-α2b) grew in 0.8 M Na_2_H_2_PO_4_, 0.8 M KH_2_PO_4_, 0.1 M Hepes pH 7.5, with diffraction to a resolution of 1.8 Å. The best crystals of complex 3 (PMAB03 (Group I)/PMAB14 (Group II)/PMAB19 (Group III)/IFN-α2b) were obtained in 0.1 M HEPES (pH 7.5) and 1.9 M (NH_4_)_2_SO_4_, with diffraction to a resolution of 4.0 Å. The crystals were flash-cooled in liquid nitrogen for data collection, with 33% (v/v) glycerol added to the crystallization solution as cryoprotectant. X-ray diffraction data were collected on beamlines PROXIMA-2A and PROXIMA-1 at the SOLEIL synchrotron (St Aubin, France). Diffraction images were integrated with autoPROC^75^ and XDS^76^ or xia2^77–79^, scaled with aimless and crystallographic calculations were performed with the CCP4 program suite^80^ (Figure S4C). The diffraction pattern of the crystal of complex 3 was strongly anisotropic and structure factor used for refinement were corrected by Staraniso.

The structures were determined by molecular replacement using Phaser^81^ and PDB model 3S9D for IFN-α2^42^ and AlphaFold models for the scFvs. The models were refined by combining real space model building in Coot^82^ with reciprocal space refinement with buster/TNT and validated with Molprobity^83,84^ (Figure S4C). The scFv models were fully built without the additional residues present in the construct (C-terminal tag and linker between heavy and light chains). Type-I IFNs are folded as a bundle of five main α-helices — A through E — with additional short helices in the loops connecting the main helices (Figure 5F). Helices A, E and a short helix connecting helices A and B (the “AB” loop”) form one side of the molecule, recognized by the IFNAR2 subunit of the receptor. The central B, C and D helices form the opposite face, recognized by IFNAR1, to make a “sandwich”-like signaling complex at the cell surface.^42,85^ Part of the IFN-α2 molecule is flexible and residues 44–49 (loop preceding Helix B) and around residue 100 (loop between helices C and D) as well as the N- and C-termini displayed no interpretable electron density in all models. The exception is loop 44–49, which is stabilized upon interaction with the 3 scFvs of complex 3. The interface analysis of the final models were performed with Pisa.^86^ Although the 4 Å resolution of the structure of complex 3 did not allow for a detailed visualization of atomic interactions within the complex, it allowed us to confidently place the 3 antibodies by molecular replacement. The epitopes of PMAB03 and PMAB19 were identical to the high confidence AF3 prediction for these two Mabs (Figure 6A). For PMAB14, AF3 could not provide a meaningful prediction, but its pose was the same as that observed independently in the 1.8 Å binary complex (compare Figures 5G and 5H). PDB, 9GVL = pmab15-Interferon alpha-2. 9GVO = pmab15-pmab14-Interferon alpha-2. 9GW5 = pmab03-pmab19-pmab14-Interferon alpha-2.

#### Structural prediction of mAb/type-I IFN complexes

All structure predictions were performed on the AlphaFold Server between September and October 2025, entering the amino-acid sequence of IFN-α2a or IFN-ϖ previously used for all epitope-binning experiments (see below), the full heavy chain and the full light chain VDJ amino-acid sequences (beginning of FR1 to end of FR4, with reversion to germline when needed for missing early or end parts of the original FR1 or FR4 sequences due to low sequencing quality at the site of primer hybridization).

IFN-α2a sequence used:

CDLPQTHSLGSRRTLMLLAQMRKISLFSCLKDRHDFGFPQEEFGNQFQKAETIPVLHEMIQQIFNLFSTKDSSAAWDETLLDKFYTELYQQLNDLEACVIQGVGVTETPLMKEDSILAVRKYFQRITLYLKEKKYSPCAWEVVRAEIMRSFSLSTNLQESLRSKE

IFN-ϖ sequence used:

CDLPQNHGLLSRNTLVLLHQMRRISPFLCLKDRRDFRFPQEMVKGSQLQKAHVMSVLHEMLQQIFSLFHTERSSAAWNMTLLDQLHTGLHQQLQHLETCLLQVVGEGESAGAISSPALTLRRYFQGIRVYLKEKKYSDCAWEVVRMEIMKSLFLSTNMQERLRSKDRDLGSS,

Default recommendations for template usage and a single random seed were used, except for a few re-expressed antibodies for which multiple seeds were tested, as recommended to maximize prediction efficiency^43^. All data and structures reported in this study correspond to model 0 of the five ranked predictions provided in the output of AlphaFold3 and corresponded to all mAbs from sorted IFN-α2 and/or IFN-ω-tetramer^+^ IgG^+^ B cells in our dataset with sequenced heavy and light chains, further filtered for reactivity to IFN-α2 and/or IFN-ω by ELISA or BLI for single-cell cultured IgG^+^ B cells.

Based on AlphaFold Server recommendations and the confidence score distribution in our dataset (Figure S5A), the AlphaFold3-predicted structures were initially classified as follows: predictions for which both IFN-α2a/heavy and light chain “chain_pair_ipTM” confidence scores >= 0.8 were considered to be of “very high confidence”, predictions with both IFN-α2a/heavy and light chain “chain_pair_ipTM” confidence scores >= 0.6, but at least one < 0.8, were considered to be “confident predictions” and the confidence level for all other predictions was considered to be “poor”. Using these criteria, “very high confidence” and “confident” predictions had global ipTM confidence scores > 0.74, with all five predictions output by AlphaFold3 being superimposable (Models 0 to 4) to RMSD values under 1Å (Table S7). Confidence scores for all selected predictions are shown in Table S2, and the frequency of confident prediction was in line with the initial model report.^60^ We favored the use of IFN-α2a/heavy and light chain “chain_pair_ipTM” confidence scores, which are probably not influenced by the well-predicted interaction between HC and LC, as these scores discriminated better between “very high confidence”/“confident” and “poor confidence” predictions. For the predicted mAbs, we obtained “very high confidence” to “confident” predictions for 23 of the 30 re-expressed IFN-α2 mAbs tested in our epitope-binning assay (Figure 5A and Figure S6). In all 23 cases, the observed competition between mAbs and IFNAR subunits was correctly predicted by AlphaFold3, including weaker competition between mAbs from Groups I and II or Groups II and III, with partial competition between PMAB18 (Group I) with PMAB02 and other Group II mAbs or between GMAB06 (Group II) and PMAB09 or other Group III mAbs (Figures 5A and 6F). We obtained “very high confidence” or “confident” predictions for three of the four mAbs for which experimental X-ray structures of the complex with IFN-α2 were determined (PMAB03, PMAB15 and PMAB19 (Figures 6A-B and S5B-C), highlighted with blue dots in Figure S5A). In all three cases, the predicted structures could essentially be superimposed on the experimental X-ray structures, with RSMD alignment scores in the 1–2 Å range (Tables S4-7), apart from the loop around Gln 46 of IFN-α2, which is only stabilized in the quaternary complex 3 (Figure 5H, S5B-C) and is accordingly not well predicted by AlphaFold in the context of a single Fv/IFN-α2 interaction. For PMAB14, the predicted epitope did not align with the experimental X-ray model (Figure 5G-I, Table S6) and the predicted global and IFN-α2a/heavy and light chain “chain_pair_ipTM” confidence scores were under 0.5 and 0.2, respectively, in all of the five independent runs performed on the AlphaFold Server with different seeds (the prediction score for one representative run is highlighted with a red dot in Figure S5A). Based on these observations, “very high confidence” and “confident” predictions were selected for downstream analysis. The data from model 0 of each prediction were used for classifications and all downstream analyses, yet the predictions were consistent, as indicated by the RMSDs of the five top predictions with the X-ray structures (Tables S5 and S6) as well as the global RMSDs obtained by the comparison among predictions (Table S7). This analysis showed that the observed RMSDs between the various predictions are not higher than those obtained for the same complex crystallized under different conditions, as shown for the IFN-α2/PMAB15 complex in Table S4.

In Figure 6B and Figure S6, the predicted structures are colored according to the predicted local distance difference test (pLDDT) confidence score for individual atoms in the prediction (AlphaFold3 “atom_plddts” full array output). The heatmaps in Figures 6A, 6F and 7A report the predicted probability of individual residues from heavy or light chains being in contact with the indicated residue in IFN-α2a or IFN-ω (8Å between the representative atom for each residue^43^; AlphaFold3 “contact_probs” full array output). In all Figures the maximum of heavy or light chain contact probabilities is shown for each residue of IFN-α2a (AlphaFold3 contact probability score). Raw contact probabilities for the heavy chain and light chain respectively are reported in Table S2. Unsupervised hierarchical clustering of all tested mAbs was performed with the maximal predicted contact probabilities for the heavy and light chains and a k value of 8 (IFN-α2a, Figure 6F) or 7 (IFN-ω, Figure 7A) for the dendextend package’s cutree() fonction. AlphaFold3-predicted structures for antibodies described in previous publications are also reported (rontalizumab, sifalimumab, and 19D11 mAbs)^19^ in Figures 6F. These structures were available and likely used for the AlphaFold3 model training. We therefore did not use these antibodies to validate our own predictions.

Representations for all predicted structures and electrostatic potential of all type-I IFNs were created with UCSF ChimeraX^87^. Helix positions and predicted IFNAR subunit contact sites were exported from the PDB sequence annotations of the corresponding IFN-IFNAR complexes (IFN-α2a: 3SE3 and IFN-ω : 3SE4,^42^). Predicted epitopes on IFN-α2a represented in Figure 6D were defined as residues with >= 0.5 (dark color) and >= 0.1 (light color) average binding probabilities, respectively, for all each epitope-binning-tested anti-IFN-α2a mAbs of indicated class. Overlaid black and purple outlines represent IFNAR2 and IFNAR1 contacts residues on IFN-α2a (PDB 3SE3). Amino-acid properties of core IFN-α2a epitopes (defined as residues with average AlphaFold contact probability with heavy or light chain of all mAbs in each main Class over 0.5) and homologous amino-acids in other type-I IFNs were calculated using the Peptides R package v2.4.6. Individual IFN recognition scores (“% of recognition by antibodies in that class”, Figure 7D) was defined as follow. mAb tested for affinity against all type-I IFNs were grouped by epitope-binning groups and each mAb, was given a score of 0 for a given IFN if its affinity was above 10^−7^ M (no detectable binding on the BLI) for that IFN, 1 if its measured affinity against that IFN was below 3nM or not above 3-fold the minimal affinity measured against all type-I IFNs for that mAb, and 0.5 for all other cases of partial loss of affinity. Individual IFN recognition scores for each Class were then defined as the average of scores from all mAbs in that Class for that IFN, further expressed as a %. Class I, II and III core epitope properties (charge (x-axis), and hydrophobicity (dot size)) are shown in Figure 7D versus re-expressed mAbs recognition score (y-axis, % of maximum binding by all antibodies in that class), with dots further colored according to binding class. All downstream analyses were performed with R v.4.4.1, using the following R packages and libraries: rjson v0.2.21, ggplot2 v.3.5.1, dendextend v1.17.1 and ComplexHeatmap v2.18.0.

#### Sequence alignment

Multiple sequence alignments were generated with Clustal Omega on the EMBL-EBI Job Dispatcher sequence analysis tools framework^88^. Visual representations of alignments were created with the Biostrings v2.70.3 and ggmsa v1.8.0 R packages (Figure S7B) or the ESPript web server ^89^ (Figures 5I, 6A, 6F and 7A). * under alignments marks an extra lysine residue in IFN-ω.

#### Illustration tool

All figures were assembled using Adobe Illustrator. The graphical abstract and Figure 1A, 2F, 2I and 4A were created with Biorender.com.

### QUANTIFICATION AND STATISTICAL ANALYSIS

Two-tailed Mann-Whitney (Figures 2A, 2H, 3A, 3C, 4D), Kruskal-Wallis (Figures 1C and 7F) and ordinary two-way ANOVA (Figure 4H) were used to compare continuous variables, and binomial tests (Figures 2B and 7C) were used to compare distributions, as appropriate. Dunn’s and Šídák's multiple comparison tests were used for multiple comparisons following Kruskal-Wallis and ordinary two-way ANOVA test, respectively. A *P*-value below or equal to 0.05 was considered statistically significant and only significant *P*-values are reported. Statistical analyses were performed in GraphPad Prism 10.6.0 (La Jolla, CA, USA), except for the two-tailed binomial tests in Figure 2B, which were performed in R with the binom.test() function further adjusted for multiple comparison according to the Benjamini & Hochberg “fdr” method in the p.adjust() function. For all statistical tests, **** *p* < 0.0001, *** *p* < 0.001, ** *p* < 0.1, * *p* < 0.05, n.s. non-significant.

## Supplementary Material

Figure S3 **Figure S3 (relates to** Figure 3). **Polyreactivity and autoreactivity of anti-IFN-α2-specific mAbs from patients with life-threatening COVID-19.** (A) Polyreactivity assessment of 32 re-expressed anti-IFN-α2-specific mAbs, defined as ELISA reactivity to insulin (left), LPS (middle) and double-stranded DNA (right). (B) Autoreactivity assessment of 32 re-expressed anti-IFN-α2-specific mAbs, defined as ELISA reactivity to HEP-2 cell-line nuclear antigens.
(C-F) Polyreactivity and autoreactivity assessment of the 11 native anti-IFN-α2-specific mAbs (blue diamonds) and their unmutated common ancestor (black diamonds), defined as ELISA reactivity to insulin (C), LPS (D) and double-stranded DNA (E) and/or HEP-2 cell-line nuclear antigens (F). The dashed horizontal line corresponds to the OD_450–620_ positive threshold of 0.09; the dashed red line with red diamond symbols corresponds to the positive control (ED38) and the white diamonds correspond to the negative control (PBS).

Figure S2 **Figure S2 (relates to** Figures 2 and 4). **AAN-I-IFN^+^ S-CoV and ITD patients harbor a highly mutated and diverse population of IFN-α2/ω-specific B cells displaying convergent V_H_/V_L_ usage**. (A) Absolute number of somatic mutations in V_L_ genes from IFN-α2/ω-specific IgG^+^ B cells sorted from ITD patients (green, *n*=163 sequences) and early sampling of AAN-I-IFN^+^ S-CoV (<2wks post hospitalization, blue, *n*=134 sequences). Bars indicate the mean±SD. The dashed line indicates *n*=2 mutations. (B) Median number of V_H_ mutations for sequences isolated from each included donors in our V(D)J dataset (n=11 AAN-I-IFN^+^ S-CoV with early sampling and n=13 ITD patients). Dots shape indicates whether more (round) or less than 15 IgH sequences (diamond) could be isolated from that donor. Each dot represents one patient; bars indicate the mean. (C) Absolute numbers of somatic mutations in the V_H_ genes of IFNα2/ω-specific B cells from the three AAN-I-IFN^+^ S-CoV and the 6 AAN-I-IFN^+^ ITD patients with more than 15 successfully sequenced cells (left). ITD patients’ sequences were further grouped based on underlying disease (APS-1 (*n*=208 sequences), Good’s syndrome (GS) and thymoma (*n*=74 sequences), partial RAG1/2 deficiency (*n*=5 sequences), and systemic lupus (SLE, *n*=8 sequences)) (right). Bars indicate the mean ± SD. The dashed line indicates *n*=2 mutations. (D) Dotplot shows V_H_/V_L_ gene pairing frequency in the repertoire of IFN-α2/ω-specific IgG^+^ B cells sorted from ITD (left) and S-CoV patients at an early (<2wks post hospitalization, middle) and late sampling (3mths to 3yrs post hospitalization, right). (E) Alpha-diversity plot calculated on the pooled repertoires of IFN-α2/ω-specific IgG^+^ B cells sorted from ITD (green line) and S-CoV patients at an early (<2wks, blue line) and late sampling (3mths-3yrs, dark grey line). (F) Shannon entropy values for the repertoires of IFN-α2/ω-specific IgG^+^ B cells sorted from individual ITD (green) and S-CoV patients at an early (<2wks, blue) and late sampling (3mths-3yrs, open circle). Only patients with more than 30 available IgH sequences were included in the analysis. (G) HCDR3 length (absolute number of amino-acids) (upper left), gravy index (upper right), net charge (bottom left) and polarity (bottom right) for all IFN-α2/ω-specific IgG^+^ B cells sorted from individual ITD (green, n=328) and S-CoV patients at an early (<2wks, blue, n=183) and late sampling (3mths-3yrs, open circle, n=101). (H) Median number of V_H_ mutations for sequences isolated from AAN-I-IFN^+^ S-CoV patients at an early (<2ks post hospitalization, n=11, blue) and/or late sampling (3mths to 3yrs post hospitalization, n=7, open circle). Dots shape indicates whether more (round) or less than 15 IgH sequences (diamond) could be isolated from that donor. Each dot represents one patient; bars indicate the mean. (I) Absolute numbers of somatic mutations in the V_H_ genes of IFN-α2/ω-specific B cells from two longitudinally sampled AAN-I-IFN^+^ S-CoV patients. Bars indicate the mean ± SD. The dashed line indicates *n*=2 mutations. (J) Lineage tree for a persisting clone (found at both time point of sampling) from patient S-CoV66, which shows signs of continued somatic hypermutation in between samplings. (A, B, H and I) Two-tailed Mann-Whitney tests. (C-G) Kruskal-Wallis tests with Dunn’s tests for multiple comparisons. ****p < 0.0001, **p < 0.01, * *p* < 0.05, n.s. non-significant.

Figure S1 **Figure S1 (relates to** Figure 1). **Experimental strategy for the selection and analysis of IFN-α2/IFN-ω-specific B cells responses in AAN-I-IFN+ patients.** (A) Patients grouping based on plasma neutralization of IFN-α2 signaling in the HD, S-CoV, and ITD cohorts included in the study (left, **see also** Table S1). Patients were considered to harbor high neutralizing anti-IFN-α2 antibody levels when displaying less than 15% (dotted red line) residual IFN signaling (expressed as a % of maximal signaling measured at the indicated IFN concentration) at a 1:10 dilution against 10ng/ml IFN-α2 (n=26 S-CoV and n=15 ITD patients). Patients were considered mid-level neutralizers if only reaching that cut-off against a 100-fold lower concentration of IFN-α2 (0.1ng/ml) (n=3 S-CoV and n=3 ITD patients). Apart from Figure S1F, both high and mid-level neutralizers were analyzed together and labeled as AAN-I-IFN^+^.
All AAN-I-IFN^+^ and most AAN-I-IFN^−^ patients were also tested for plasma neutralization of IFN-ω (middle) and IFN-β (right). Only seven plasma samples from AAN-I-IFN^−^ S-CoV patients were available for testing with 10 ng/ml IFN-α2/ω and 1ng/ml IFN-β. Each dot represents a patient; the bars indicate the median. (B) Gating strategy used for identification of the main B-cell subpopulations present in the patients’ PBMCs. B cells were defined as live Decoy^−^ CD19^+^ CD3^−^CD14^−^ singlets, with antibody-secreting cells (ASCs) removed by the exclusion of CD27^+^CD38^hi^ cells. Non-ASCs were analyzed according to CD27 expression (IgD^−^CD27^+^ memory B cells (MBCs) versus IgD^−^CD27^−^ double-negative B cells (DNs)) and split into further subpopulations according to CD11c and CD21 expression (DN2: CD27CD11c^+^CD21^low^; other DNs: CD27^−^CD11c^−^CD21^low/+^; activated B cells (ABC): CD27^+^CD11c^+^CD21^low^ and resting MBCs (rMBC): CD27^+^CD11c^−^CD21^low/+^). Representative overlayed staining for CD19, CD71, T-bet and CXCR5 in DN2, other DNs, ABCs and rMBCs is further shown below. (C-D) Proportion of CD19^+^CD27^+^CD38^hi^ASCs among CD19^+^ cells (C) and proportion of CD27^−^IgD^−^CD21^low/-^CD11c^+^ double-negative 2 (DN2) amongst non-ASCs B cells (D) in HD (gray), AAN-I-IFN^−^ (orange) or AAN-I-IFN^+^ S-CoV (blue), and AAN-I-IFN^+^ ITD patients (green). ITD patients were further separated based on underlying disease in the right panel of (D). Each dot plot represents one patient; bars indicate the median. (E) IFN-α2 and IFN-ω tetramer staining and validation strategy. IFN-α2/IFN-ω-specific IgG^+^ B cells were stained on the basis of a combination of IFN-α2 PE/BV421 and IFN-ω BV421/BV711 tetramers and IFN-α2 and/or IFN-ω-tetramer^+^ IgG^+^ B cells were single-cell sorted and *ex vivo* culture for differentiation into ASCs on hCD40L^lo^ feeder cells (Top). After three to four weeks, supernatants were tested with an in-house ELISAs (bottom) to determine the true purity of the sorted single-cells according to the tetramer staining strategies tested (panel 1: IFN-α2 PE/APC, with representative staining in IgG^+^ B cells shown in the left panel, panel 2: IFN-α2 BUV395/BV421 and panel 3: IFN-α2 PE/BV421 and IFN-ω BV421/BV711). All supernatants with an optical density (OD) ≥0.09 were considered IFN-α2-specific. Overall purity is shown on each graph. Panel 3 gave the highest overall purity (>90%) and was used thereafter for the staining of all the remaining samples in this study. (F) Frequencies of IFN-α2-specific IgG^+^ B cells in HD (gray, *n*=14), AAN-I-IFN^−^ S-CoV (orange, *n*=11), AAN-I-IFN^+^ S-COV (blue) and ITD patients (green) (left), with the latest two groups being further separated on the basis of plasma neutralization levels as defined in Panel S1A. As in D, ITD patients were also separated based on underlying disease (APS-1 (*n*=6), NF-kB2 deficiency (*n*=4), partial RAG1/2 deficiency (*n*=2), Good’s syndrome (GS) and thymoma (*n*=5) and systemic lupus (SLE, *n*=3)) (right). Each dot represents a patient; bars indicate the median. (C-D) Kruskal-Wallis tests with Dunn’s tests for multiple comparisons. **** *p* < 0.0001, *** *p* < 0.001, **p < 0.01.

Figure S4 **Figure S4 (relates to** Figure 5). **Structural basis of IFN-α2 binding and neutralization in patients with life-threatening COVID-19.** (A) Heatmap of competitive binding to IFN-α2 for the first 14 re-expressed Abs in our dataset, all isolated from sorted IFN-α2-specific IgG^+^ B cells from S-CoV patients, as well as published anti-IFN-α2 monoclonal antibodies rontalizumab ^40^ and sifalimumab ^41^ and the high-affinity IFNAR subunit (IFNAR2). All mAbs and IFNAR2 were tested against each other in both directions using a classical epitope-binning sandwich assay approach (see Figure 5B). Rows represent the first mAb/receptor subunit loaded (biosensor-bound mAb/receptor) and columns represent the second antibody/receptor subunit loaded (competing mAb/receptor). Row names and Col names of representing members used in Figure 5A and 5C for each of the three major identified epitope groups are further highlighted (Group I, green; Group 2, red; Group III, amber). (B) Neutralization of IFN-α2 for all tested re-expressed mAbs grouped by epitope group (half-maximal inhibitory concentration) relative to the measured off-rate (k_off_, 1/s) against IFN-α2. (C) Data collection and refinement statistics for the three complexes described as part of Figure 5F-H. (D-E) Heavy chain (D) and light chain (B) alignments with the closest gene and allele from the IMGT V domain directory for all members of the three complexes described as part of Figure 5F-H. IMGT-defined framework (FR1, FR2, FR3 and FR4) and complementarity-determining regions (CDR1, CDR2 and CDR3) are indicated on top of each alignment. Observed contacts with IFN-α2 (see Figure 5F-H) are shown above involved amino-acids in each sequence (H: Hydrogen bond, S: salt bridge, *: simple contact). Germline amino-acid residues are indicated below mutated residues in each sequence. The lines for each alignment are colored according to epitope-binning group for corresponding mAb.

Figure S5 **Figure S5 (related to** Figures 6 and 7). **Quality measurements for AlphaFold3 predictions.** (A) AlphaFold3’s global and IFN-α2a/heavy and light chain_pair_ipTMs for all IgH/IgL pairs tested from isolated IFN-α2-specific B cells (top left, n=317) and IFN-ω-specific B cells (top right, n=75) and all pairs with predictions considered to be confident or of very high confidence against IFN-α2a (bottom left, n=167) or IFN-ω (bottom right, n=44) (**see** Methods). Prediction scores for all four IgH/IgL pairs for crystallized complexes of anti-IFN-α2 monoclonal antibodies interacting with IFN-α2 (see also Figure 5) are highlighted in blue when confident or very high confidence (PMAB03, PMAB15 and PMAB19) or red when of “poor” confidence (PMAB14). (B-C) C*α*-carbon root-mean square deviation (RMSD) upon superimposing the IFN-*α*2 moiety only of selected complexes and visualized using the cartoon putty representation within PyMOL^90^ in which the ribbon thickness is proportional to the RMSD value and using a color ramp from 0 to 10Å RMSD according to the color bar. (B) The three experimental structures of the PMAB15 scFv in complex with IFN-*α*2. Complexes 1A and 1B correspond to two independent scFv/ IFN-*α*2 complexes in the asymmetric unit of crystal 1 (complex 1 in Figure 5F) while complex 2 corresponds to the ternary complex of IFN-*α*2 with the scFvs of PMAB15 and PMAB14 (complex 2 in Figure 5G). The V_L_ region of the PMAB15 scFv is altered by crystal packing contacts in complex 2, resulting in high RSMD values. The overall RMSD values are listed in Table S4. (C) Comparison of the X-ray structure of three different scFv/IFN-*α*2 complexes with the corresponding AF3 prediction of highest confidence. The PMAB15/IFN-*α*2 complex is displayed in the same orientation as in B), whereas the other two are displayed with the heavy and light chain of the antibody roughly oriented as in the PMAB15 complex, resulting in different orientations of the IFN-*α*2 in the complex with PMAB19, which binds to a different antigenic site. Not that the loop around Gln 41, which has high RMSD values, is mobile and displays no electron density in most of the experimental structures. The structures of the PMAB19 and PMB03 complexes used here correspond to the X-ray structure in complex 3 (Figure 5H) at 4Å resolution, while that of PMAB15 corresponds to complex 1 (Figure 5F) at 2.05Å resolution. In the case of the fourth scFv/ IFN-*α*2 complex for which we have an experimental structure, corresponding to PMAB14 (present in complexes 2 and 3 (Figure 5G-H), Alphafold3 could not confidently predict its binding mode (see RMSD values in Table S5) and comparison between the low confidence predictions and observed structures of this complex are therefore not represented in this Figure. The plotted RMSD values correspond to those in Figure S6, using the AF3 prediction with highest score in each case (see also Tables S4-7 for detailed RMSDs upon superposition). (D and E) Histograms showing the accuracy of AlphaFold3-predicted structures for all IgH/IgL pairs against IFN-α2a (D) or IFN-ω (E) analyzed according to V_H_ gene usage. The absolute number of IgH/IgL pairs tested for each V_H_ is indicated above each bar. (F) Histograms showing the accuracy of AlphaFold3-predicted structures for all re-expressed and epitope-binning-tested monoclonal Abs against IFN-α2a (left) or IFN-ω (right), pooled by epitope-binning group. (G) Histograms showing the accuracy of AlphaFold3-predicted structures for all IgH/IgL pairs against IFN-α2a (left) or IFN-ω (right), analyzed according to junction length. In (F-G), the absolute number of IgH/IgL pairs tested in each bin is indicated above each bar (see also Tables S2).

Figure S7 **Figure S7 (related to** Figure 7). **Epitope-related constraints on cross-reactivity and polyreactivity selection in type-I IFN-specific auto-Abs.** (A) Donut plots showing the distribution between the three main structural IFN-ω-binding classes predicted by AlphaFold3 for all IgH/IgL pairs from S-COV and ITD patients for which predictions against IFN-ω were “confident” to “very high confidence”. The total number of IgH/IgL pairs retained for the final analysis is indicated in the center of each donut plot. (B) Alignment of all IFN-α subtypes and IFN-ω. The boxes on the plot represent the resolved positions of helices on IFN-α2 and IFN-ω (PDB 3SE3 and 3SE4). (C-D) Surface representation of representative type-I IFNs colored according to electrostatic potential, focusing on the core Class II (C) and Class III epitope (D) sides of IFN-α2 (contours of each epitope are overlayed on IFN-α2a surface representation). Net charge values for the core Class II (C) and Class III epitope (D) of each IFN are indicated at the bottom of each representation. (E) Graphs showing HCDR3 gravy index (far left), polarity (left) and percent of bulky (middle), aliphatic (right) or aromatic amino-acids (far right) for all 163 IFN-α2-specific mAbs whose AlphaFold prediction felt in one of the 3 main binding Class identified. (F) Graphs showing HCDR3 gravy index (left) and polarity (right) for all 163 IFN-α2-specific mAbs whose AlphaFold prediction felt in one of the 6 dominant binding sub-Class identified. (G) Graph showing HCDR3 length (absolute number of amino-acids) (left), net charge (middle) and percent of acidic amino-acids (right) for all 44 IFN-α2/ω-specific mAbs whose AlphaFold prediction felt into Class III, further grouped according to their reactivity to IFN-α2 and IFN-ω. Cross-reactive mAbs are included in the IFN-α2-reactive group. (E-F) Kruskal-Wallis tests with Dunn’s tests for multiple comparisons and (G) Two-tailed Mann-Whitney tests. **** p < 0.0001, *** p < 0.001, **p < 0.01, *p < 0.05, n.s. non-significant.

Figure S6 **Figure S6 (related to** Figures 6 and 7). **AlphaFold3 predictions for re-expressed and epitope-binning-tested anti-IFN-α2/-ω monoclonal antibodies isolated from patients with life-threatening COVID-19.** (A-F) All structures predicted with confidence or a very high level of confidence by AlphaFold3 for re-expressed monoclonal Abs in interaction with IFN-α2a (*n*=23) or with IFN-ω (*n*=3), and further separated by identified epitope-binning group (I, green (A); II, red (B); III, yellow (C); I (IFN-ω only), dark green (E); other (IFN-ω only), brown (F)). One additional representative, but not re-expressed, mAbs predicted to target the connecting loops at the front of IFN-α2a is shown (grey, (D)). Global and local (IgH/IFN-α2 or IFN-ω and IgL/IFN-α2 or IFN-ω) ipTM scores are shown, together with the sequenced V_H_/V_L_ genes. AlphaFold3-predicted structures are colored according to the pLDDT confidence score of individual residues (see also Table S2). *Note that all these mAbs were isolated from S-CoV patients except one, GMAB07, isolated from the spleen of an AAN-I-IFN*^*+*^*AAb organ donor (see* Tables S1 and S3).

Table S1 Table S1: Patients’ information, related to Figure 1.

Table S3 Table S3: Excel file containing additional data too large to fit in a PDF, related to the re-expressed IFN-α2-specific monoclonal antibodies, related to Figures 3, 5, 6 and 7.

Table S4

Table S5

Table S6

Table S7

Table S2 Table S2: Excel file containing additional data too large to fit in a PDF, related to the repertoire and function of isolated IFN-α2-specific patients’ B cells, related to Figure 2, 4, 6 and 7.

Document S1: Full Details of Consortium Authors

## Figures and Tables

**Figure 1.**
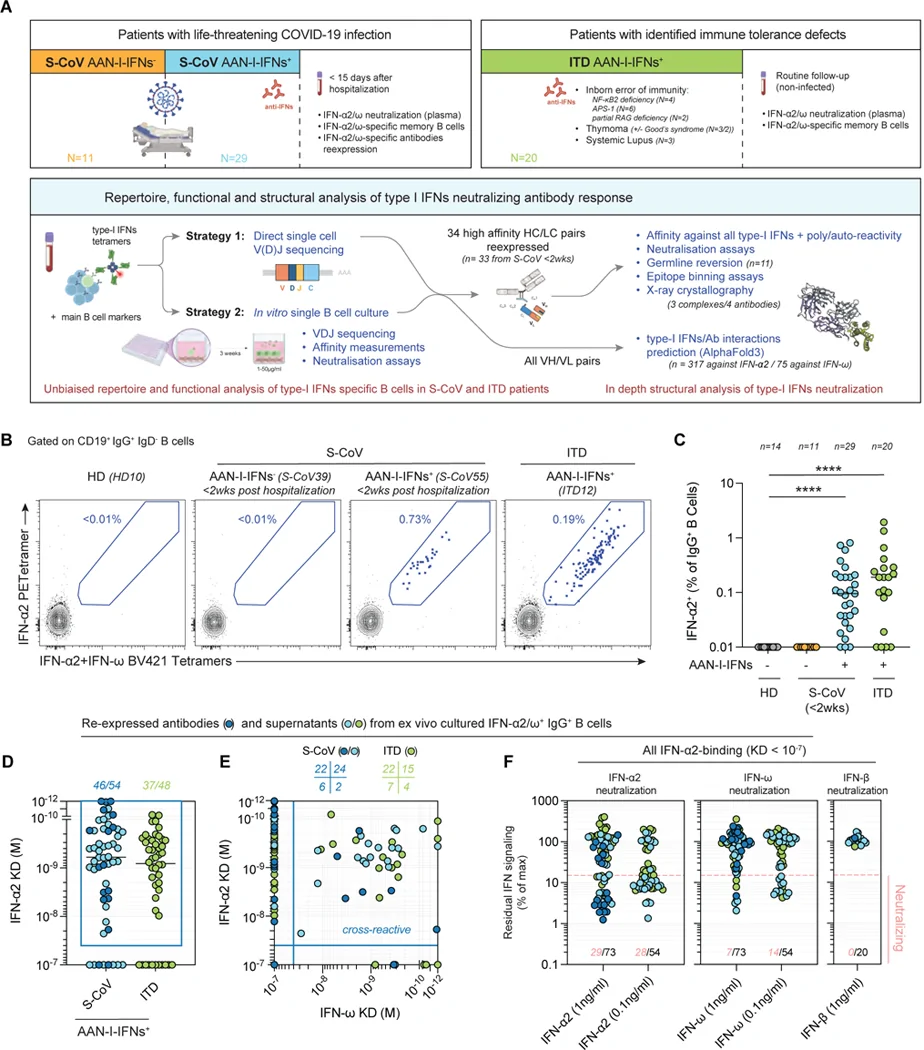
High-affinity type-I IFN-specific B cells with neutralizing activity in life-threatening COVID-19 and ITD patients. **(A)** Study cohorts and analytical workflow. (B-C) IFN-α2/ω staining and frequencies of IFN-α2-specific cells amongst CD19^+^IgG^+^IgD^−^ B cells in PBMCs from healthy donors, recently hospitalized patients with life-threatening COVID-19 (early S-CoV ± AAN-I-IFN), and ITD patients. (D-F) Affinity for IFN-α2a and IFN-ω and neutralization of IFN-α2, IFN-ω, or IFN-β by re-expressed monoclonal antibodies and ELISA-validated single-cell culture supernatants from sorted IFN-α2/ω^+^ IgG^+^ B cells (early AAN-I-IFN^+^ S-CoV and ITD). Only IFN-α2-binding antibodies (KD < 10⁻⁷ M) were tested for neutralization. Bars indicate medians (C-D). Statistical tests detailed in Methods. See also Fig. S1 and Table S1.

**Figure 2.**
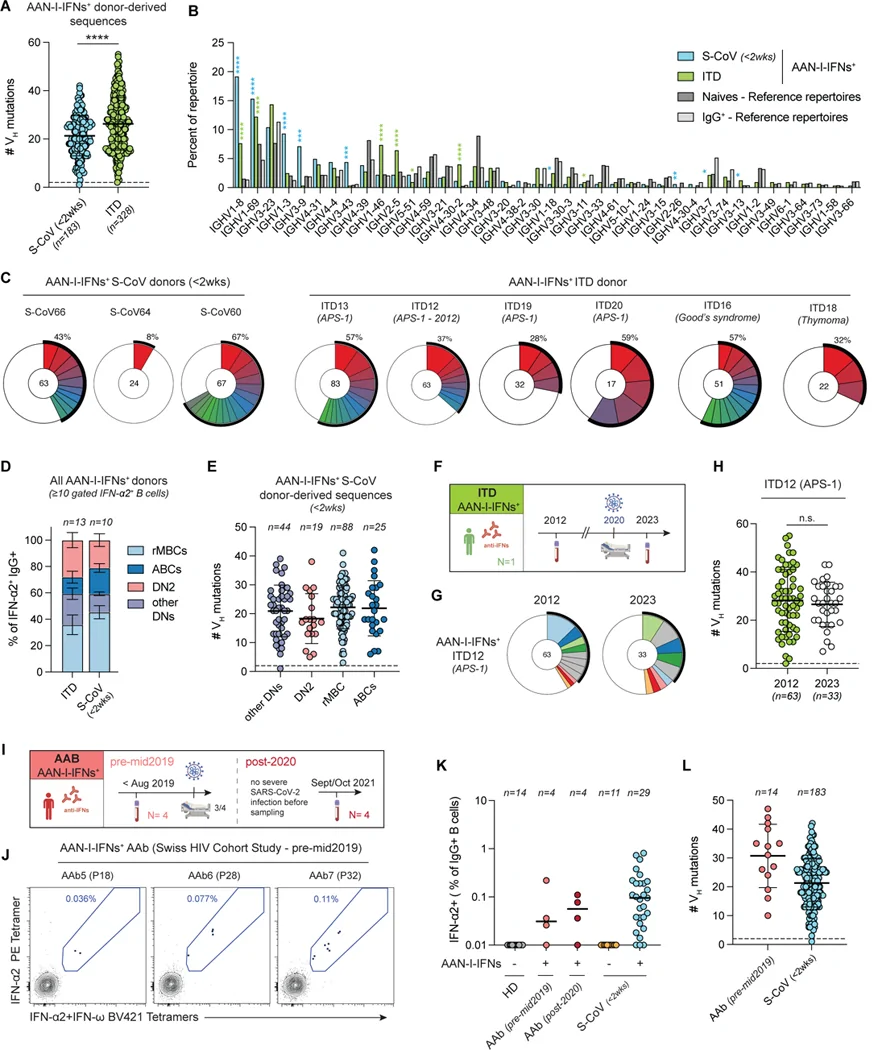
Repertoire, phenotype, and pre-existence of IFN-α2-specific B cells in life-threatening COVID-19 patients. (A-B) V_H_ somatic mutation burden and V_H_ gene usage in IFN-α2/ω-specific IgG^+^ B cells from ITD and early AAN-I-IFN^+^ S-CoV patients, compared with naive and IgG^+^ B cells from public datasets. (C) Clonal distributions of IFN-α2/ω-specific IgG^+^ B cells in AAN-I-IFN^+^ S-CoV and ITD patients. (D-E) Distribution and V_H_ mutation burden of IFN-α2-specific IgG^+^ B cells across major IgD^−^ B cell subsets. (F-H) Longitudinal sampling, clonal persistence, and V_H_ mutation mutation burden of IFN-α2/ω-specific IgG^+^ B cells in an ITD patient sampled before and after severe COVID-19. Colored slices indicate persistent clones; white and gray denote singlets and non-persisting expanded clones. (I-L) Sampling timeline, representative staining, frequencies, and V_H_ mutation burden of IFN-α2/ω-specific IgG^+^ B cells in individuals with age-associated anti-type-I IFN autoantibodies sampled pre-pandemic or in 2021. Bars indicate median (K), mean±SEM (D) or mean±SD (all others); dashed lines mark two V_H_ mutations. Statistical tests detailed in Methods. See also Fig. S2 and Table S2.

**Figure 3.**
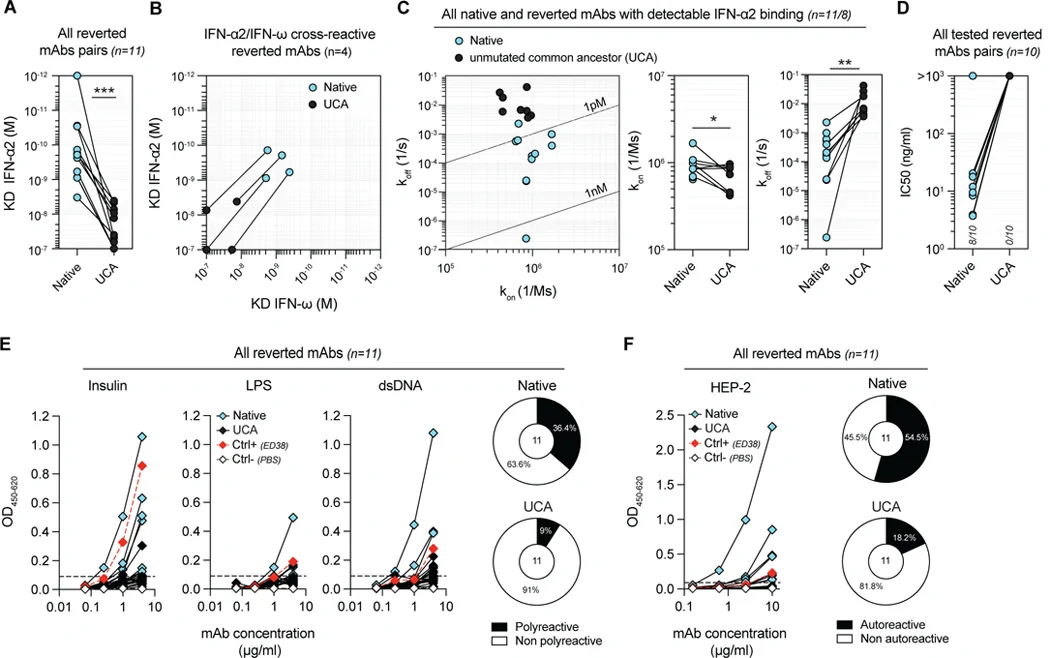
Affinity maturation and selection of poly/autoreactivity in high-affinity anti-IFN-α2 autoantibodies. (A-D) Affinity for IFN-α2 or IFN-ω/IFN-α2, kinetic rate constants for IFN-α2 binding, and IFN-α2 neutralization (IC₅₀) by native re-expressed monoclonal antibodies and their unmutated common ancestors (UCAs). (E-F) Polyreactivity and autoreactivity of native antibodies and paired UCAs; OD₄₅₀–₆₂₀ threshold = 0.09, with ED38 and PBS as positive and negative controls. Donut plots show frequencies of auto-/polyreactive antibodies. Statistical tests detailed in Methods. See also Fig. S3 and Table S3.

**Figure 4.**
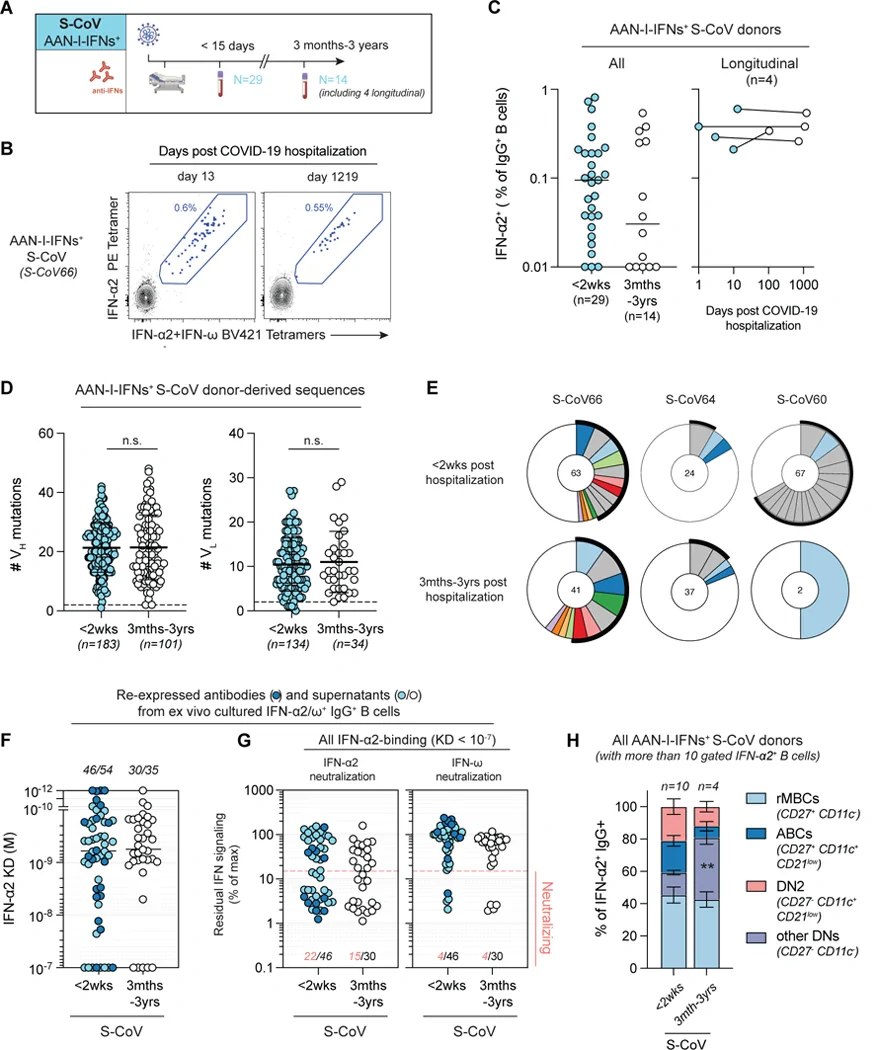
Long-term stability of the anti-IFN-α2/ω B cell response after SARS-CoV-2 infection. (A-E) Sampling timeline, representative IFN-α2/ω staining, frequencies, V_H_ and V_L_ somatic mutation burden and clonal evolution of IFN-α2–specific CD19^+^IgG^+^IgD^−^ B cells in AAN-I-IFN^+^ S-CoV patients sampled early after hospitalization or during follow-up. Colored slices indicate persistent clones; white and gray denote singlets and non-persisting expanded clones. (F-G) Affinity for IFN-α2 and neutralization of IFN-α2 or IFN-ω by re-expressed monoclonal antibodies and culture supernatants from sorted IFN-α2/ω-specific IgG^+^ B cells at early and late time points. (H) Distribution of IFN-α2/ω-specific IgG^+^ B cells across major IgD^−^ B cell subsets at early and late time points. Bars indicate medians (C and F), mean±SEM (H) or mean±SD (D); dashed line marks two V_H_ mutations. Statistical tests detailed in Methods. See also Fig. S2 and Table S2.

**Figure 5.**
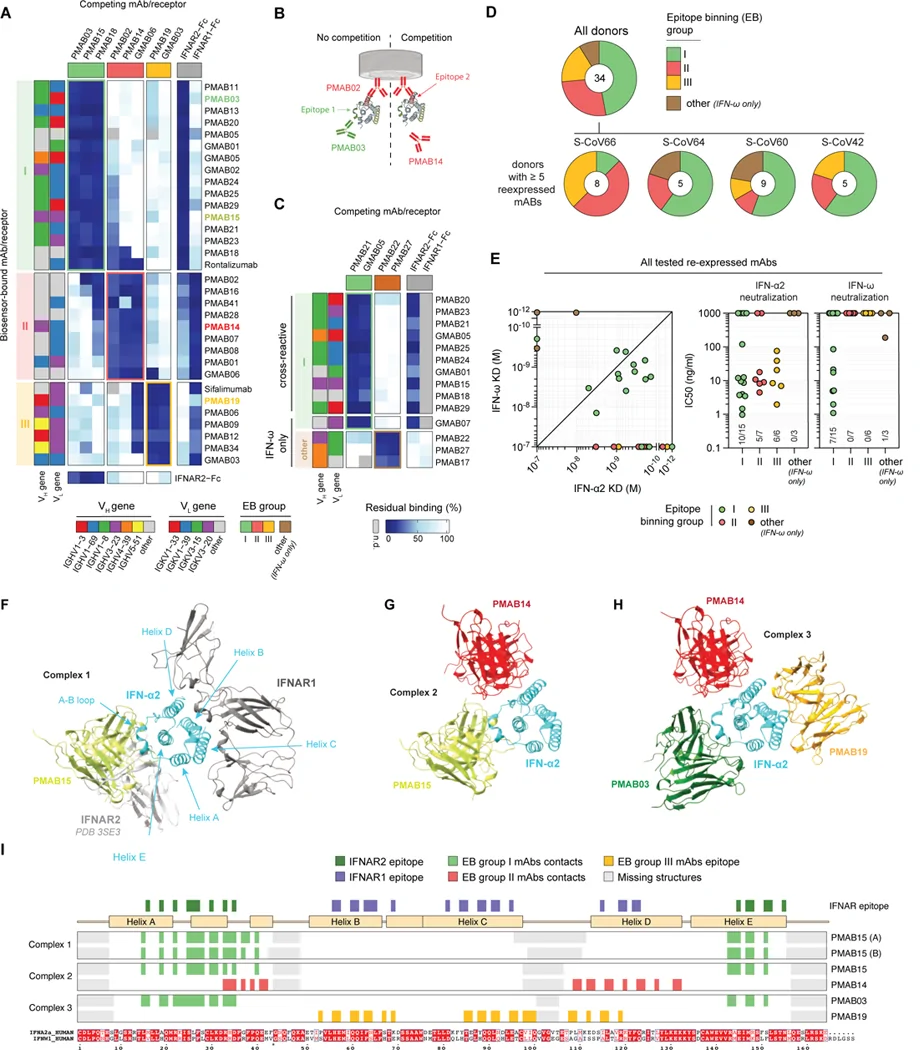
Structural basis of IFN-α2 binding and neutralization in life-threatening COVID-19. (A-C) Epitope-binning strategy and competitive binding heatmaps for IFN-α2- and IFN-ω-specific monoclonal antibodies against a reference set of anti-IFNs antibodies (see Figure S4A) or IFNAR1/IFNAR2; row annotations indicate dominant VH/VL gene usage. (D) Epitope-binning group distribution across all tested antibodies and within individual S-CoV patients. (E) Affinity for IFN-α2a and IFN-ω and neutralization grouped by epitope-binning group. (F-I) X-ray structures of binary (with overlaid IFNAR1/IFNAR2 structure, PDB 3SE3), ternary, and quaternary antibody-IFN-α2b complexes and heatmap of observed antibody-IFN-α2 contacts; IFNAR1/2 contact residues and IFN-α2 structural elements indicated in (I). See also Fig. S4 and Table S3.

**Figure 6.**
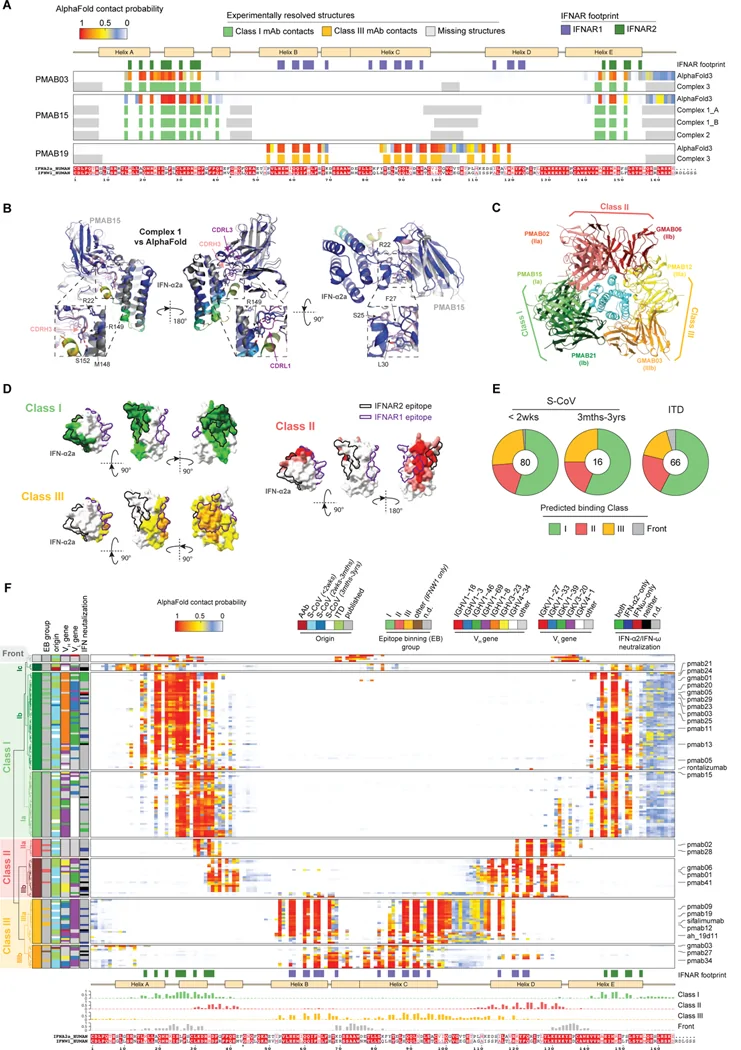
Structural diversity of IFN-α2 epitopes targeted in life-threatening COVID-19 and ITD patients. (A) Heatmap of observed (X-ray) versus predicted (AlphaFold3) contacts for anti-IFN-α2 antibodies with paired X-ray structures, colored by epitope-binning group (observed) or AlphaFold3 contact probability (predicted). (B) Superposition of predicted and resolved PMAB15/IFN-α2 structures, with CDRs highlighted and key interacting residues shown. (C-D) Representative AlphaFold-predicted structures and structural epitopes on IFN-α2a for each major anti-IFN-α2 class; IFNAR1/2 epitopes indicated. (E-F) Distribution of structural IFN-α2a-binding classes by sample origin and heatmap of predicted contacts for all IFN-α2a-specific IgH/IgL pairs with confident/high-confidence predictions (n = 167) and three published anti-IFN-α2 antibodies. Row annotations indicate epitope-binning group, sample origin, dominant V_H_/V_L_ genes, and neutralization potential. Colored histograms below show mean binding probabilities for each main classes. See also Figs. S5–S6.

**Figure 7.**
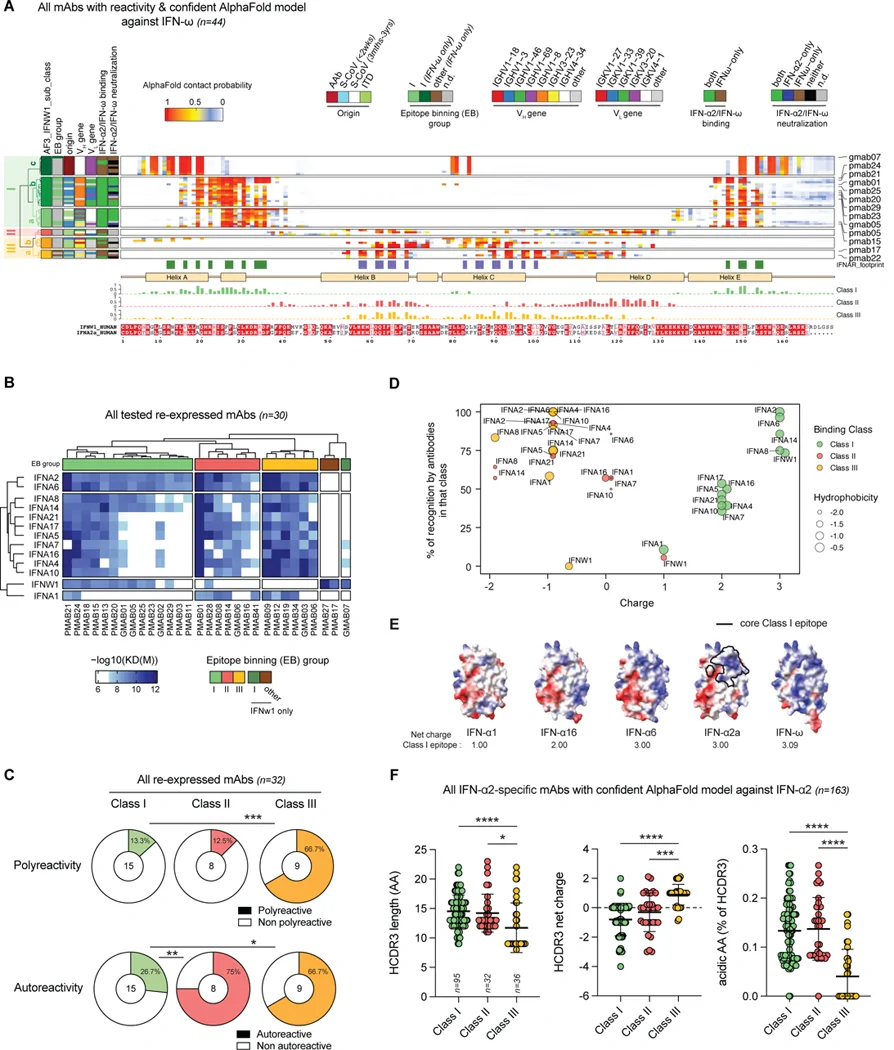
Epitope-related constraints on cross-reactivity and poly-/autoreactivity in type-I IFN-specific autoantibodies. (A) Heatmap of predicted IFN-ω contacts for all IFN-ω-specific IgH/IgL pairs with confident/high-confidence AlphaFold3 predictions (n = 44); row annotations indicate epitope-binning group, sample origin, V_H_/V_L_ usage, and binding/neutralization potential to IFN-α2a and IFN-ω. Colored histograms below show mean binding probabilities for each main classes. (B–C) Heatmap of log₁₀ KD values against IFN-α subtypes/IFN-ω (B) and poly-/autoreactivity (C) of re-expressed antibodies, grouped by epitope-binning group. (D) Core Class I-III epitope properties (charge, hydrophobicity) versus antibody recognition score (% of maximum binding within class). (E) Surface electrostatic potential of representative type-I IFNs focused on Class I IFN-α2 epitope (overlaid on IFN-α2). (F) HCDR3 length, net charge, and fraction of acidic residues for IFN-α2-specific antibodies grouped by main predicted binding class. Statistical tests detailed in Methods. See also Fig. 3 and S7.
