# Supplementary material for: Affinity-matured B cell responses neutralizing type-I interferons underlie severe viral infections
Source: Cell. Author manuscript; Available in PMC 2026 Oct 6. (PMC13639993; doi:10.1016/j.cell.2026.04.013)

Figure S1 (related to Figure 1):

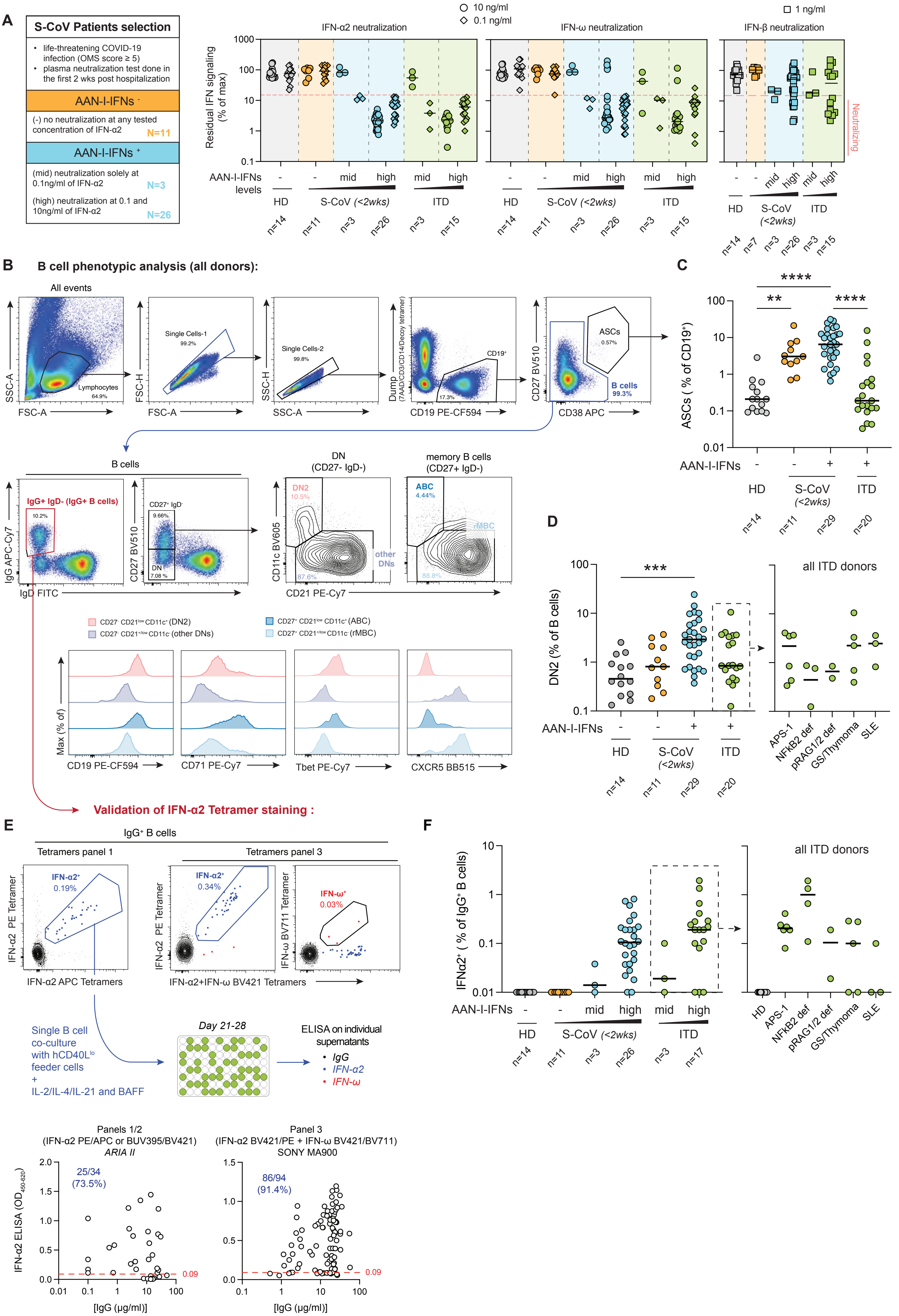

Supplement: Supplementary file 1 [file NIHMS2205108-supplement-Figure_S1.pdf]
