# Supplementary material for: Affinity-matured B cell responses neutralizing type-I interferons underlie severe viral infections
Source: Cell. Author manuscript; Available in PMC 2026 Oct 6. (PMC13639993; doi:10.1016/j.cell.2026.04.013)

Figure S2 (related to Figures 2 and 4):

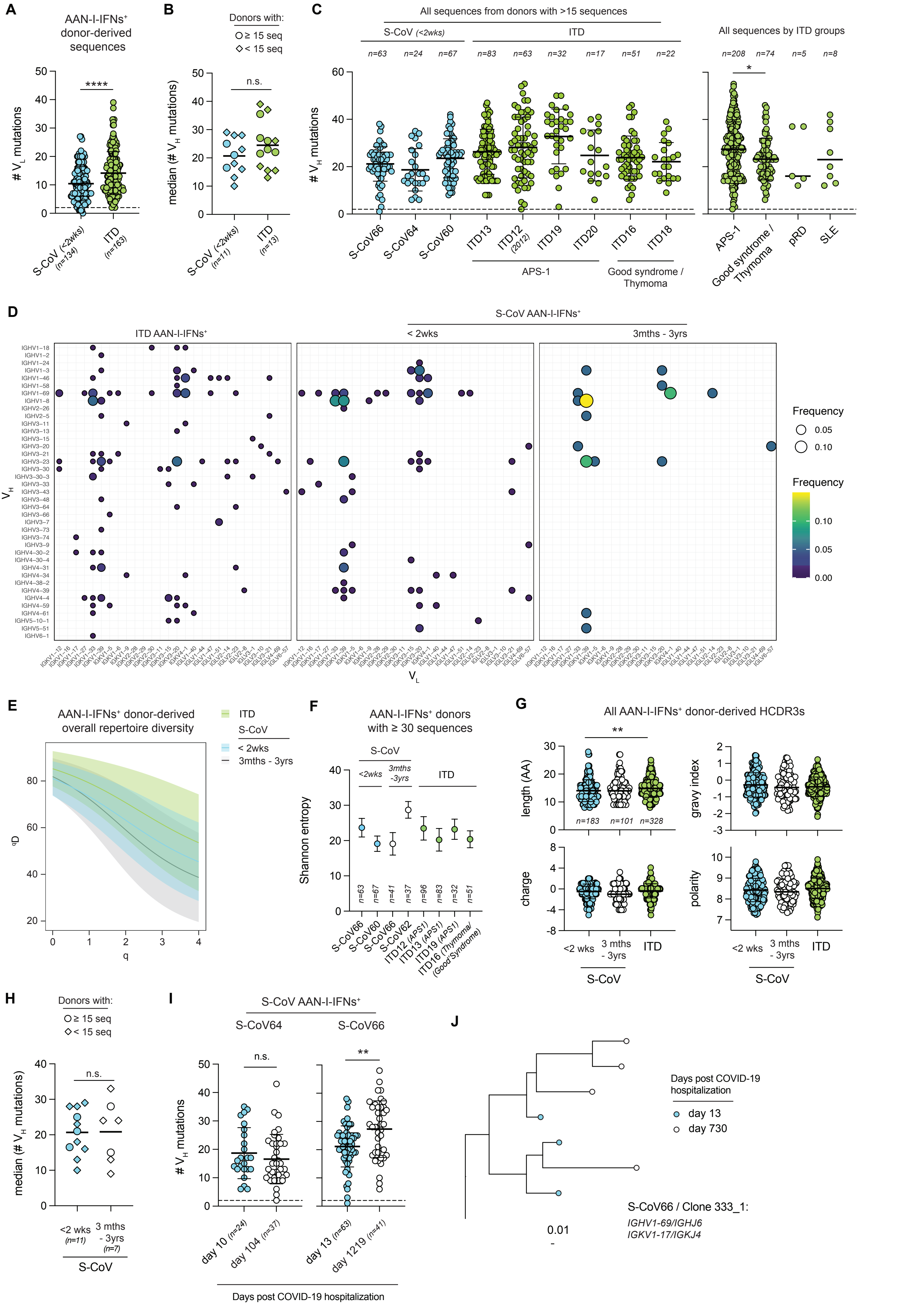

Supplement: Supplementary file 2 [file NIHMS2205108-supplement-Figure_S2.pdf]
