# Supplementary material for: Affinity-matured B cell responses neutralizing type-I interferons underlie severe viral infections
Source: Cell. Author manuscript; Available in PMC 2026 Oct 6. (PMC13639993; doi:10.1016/j.cell.2026.04.013)

Figure S4 (related to Figure 5):

A

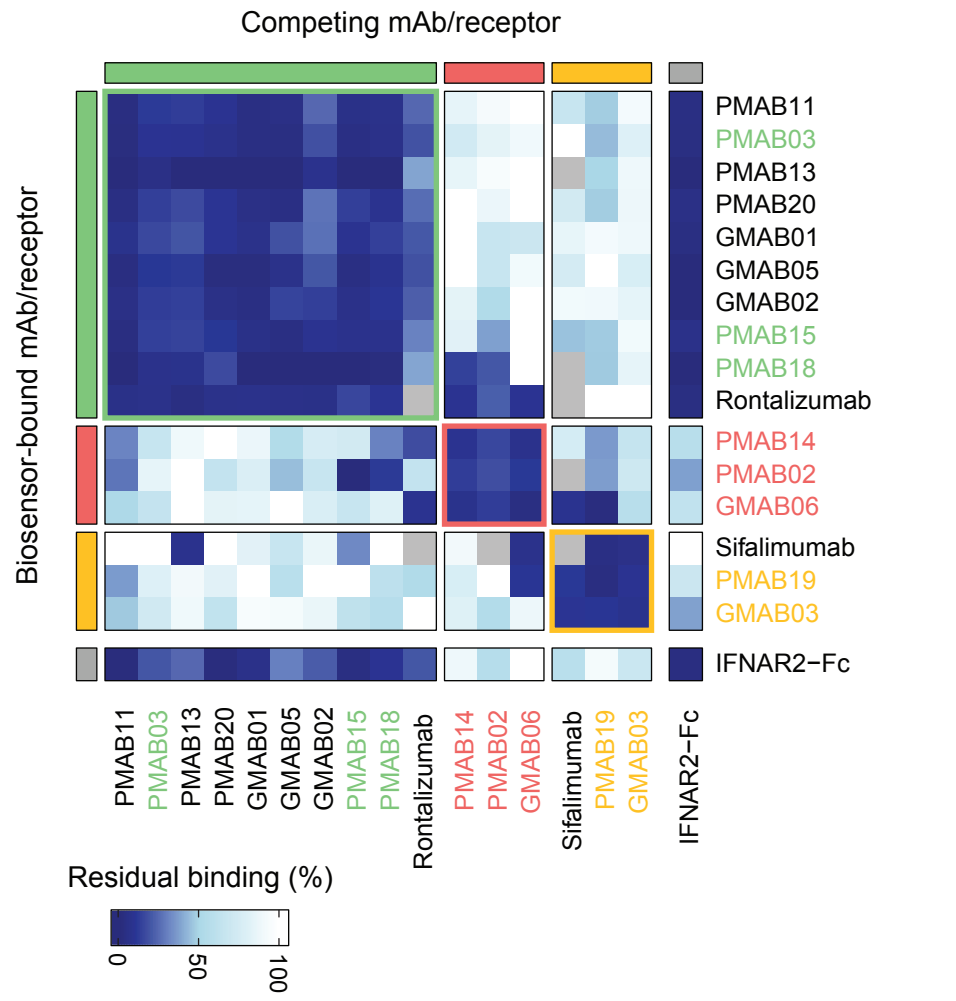

B

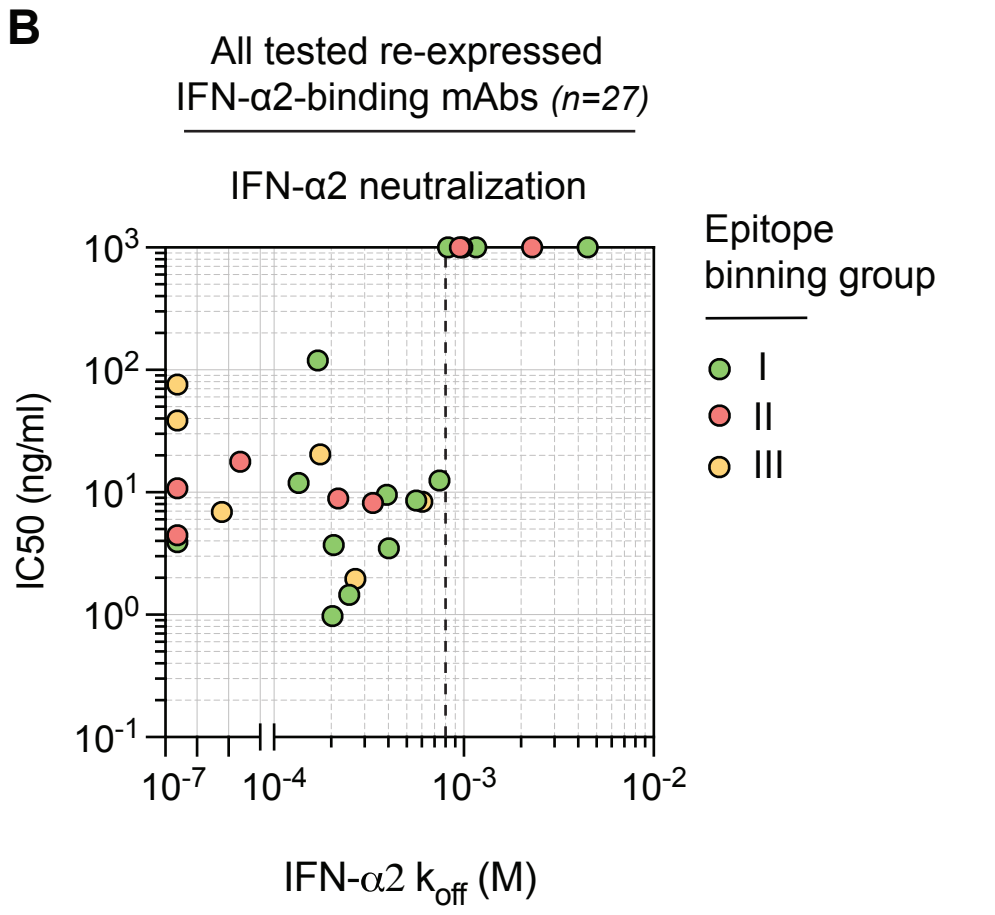

C

Data collection and Refinement statistics

IFN-α2 in complex with	PMAB15	PMAB15+PMAB14	PMAB14+PMAB03+PMAB19
PDB accession code	9GVL	9GVO	9GW5
**Data collection**	PROXIMA-2A	PROXIMA-1	PROXIMA-2A
Space group	P 1 21 1	P 31 2 1	P 65 2 2
Unit cell parameters			
a,b,c (Å)	66 – 84 - 70	113 – 113 - 190	166 – 166 - 190
α,β,γ (°)	90 – 113.8 - 90	90 – 90 - 120	90 – 90 - 120
Resolution (Å)	65-2.0 (2.041-2.006)	50-1.81 (1.84-1.81)	150-4.00 (4.128-4.000)
Total observations	236673 (11701)	5859460 (106659)	1064307 (84570)
Unique reflections	46986 (2310)	105167 (5103)	13477 (1038)
Completeness (%)	100.0 (99.7)	100 (98.5)	98.7 (86.9)
Redundancy	5.0 (5.1)	55.7 (20.9)	79.0 (81.5)
<I/s>[b]	3.6 (0.6)	17.1 (0.5)	8.8 (1.1)
R_{merge} (%)	0.202 (2.611)	0.146 (5.184)	0.781 (8.869)
R_{meas} (%)	0.225 (2.902)	0.147 (5.313)	0.786 (8.923)
R_{pim} (%)	0.098 (1.667)	0.019 (1.159)	0.128 (1.379)
CC(1/2)	0.991 (0.373)	1.00 (0.353)	0.996 (0.686)
**Refinement**			
Resolution (Å)	15-2.01 (2.02-2.01)	15-1.81 (1.82-1.81)	25-4.00 (4.07-4.00)
Number of reflections	46627 (933)	104583 (1984)	13185 (396)
Number of Rfree reflections	2428 (49)	5205 (108)	645 (17)
Rfactor (%)	21.43 (38.80)	0.1925 (0.3497)	29.43 (32.36)
Rfree (%)	26.96 (37.05))	0.2250 (0.2150)	29.56 (30.61)
Number of protein atoms	5645	4677	6368
Number of waters	287	384	0
Root mean square deviations			
Bond lengths (Å)	0.010	0.009	0.006
Bond angles (°)	0.91	0.94	0.84
Ramachandran fav/out (%)	98.2/0.0	98.14/0.17	95.82/0.61

D

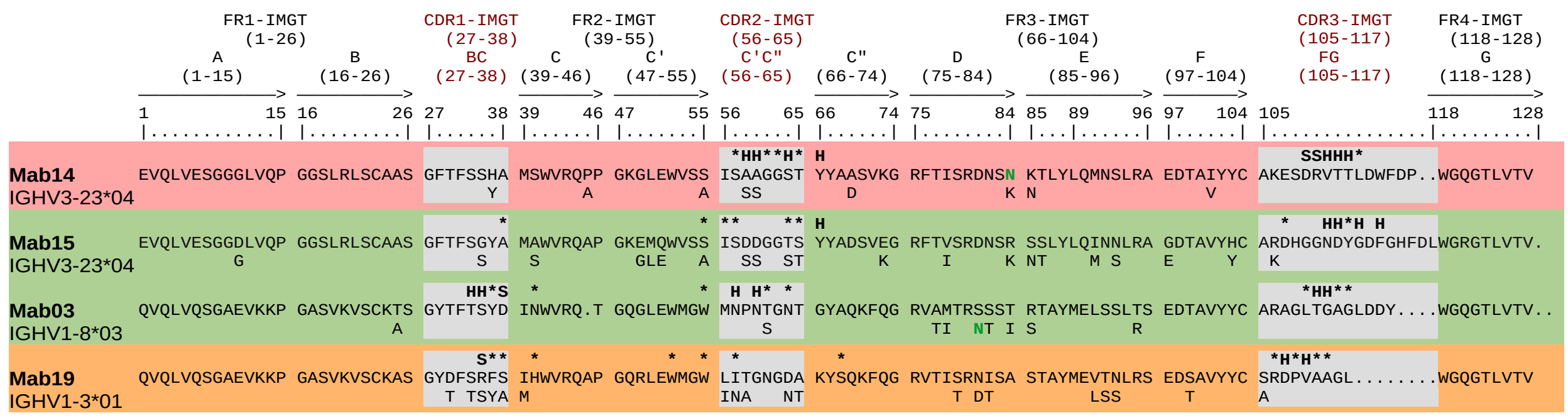

E

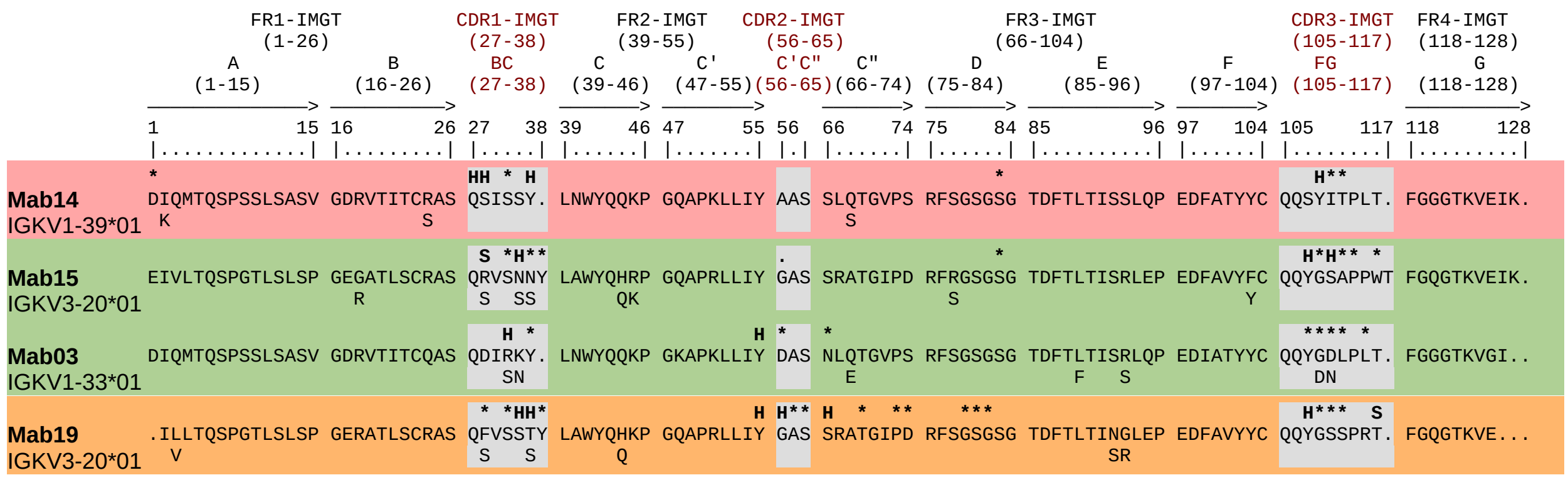

Supplement: Supplementary file 4 [file NIHMS2205108-supplement-Figure_S4.pdf]
