# Supplementary material for: Affinity-matured B cell responses neutralizing type-I interferons underlie severe viral infections
Source: Cell. Author manuscript; Available in PMC 2026 Oct 6. (PMC13639993; doi:10.1016/j.cell.2026.04.013)

Figure S5 (related to Figures 6 and 7):

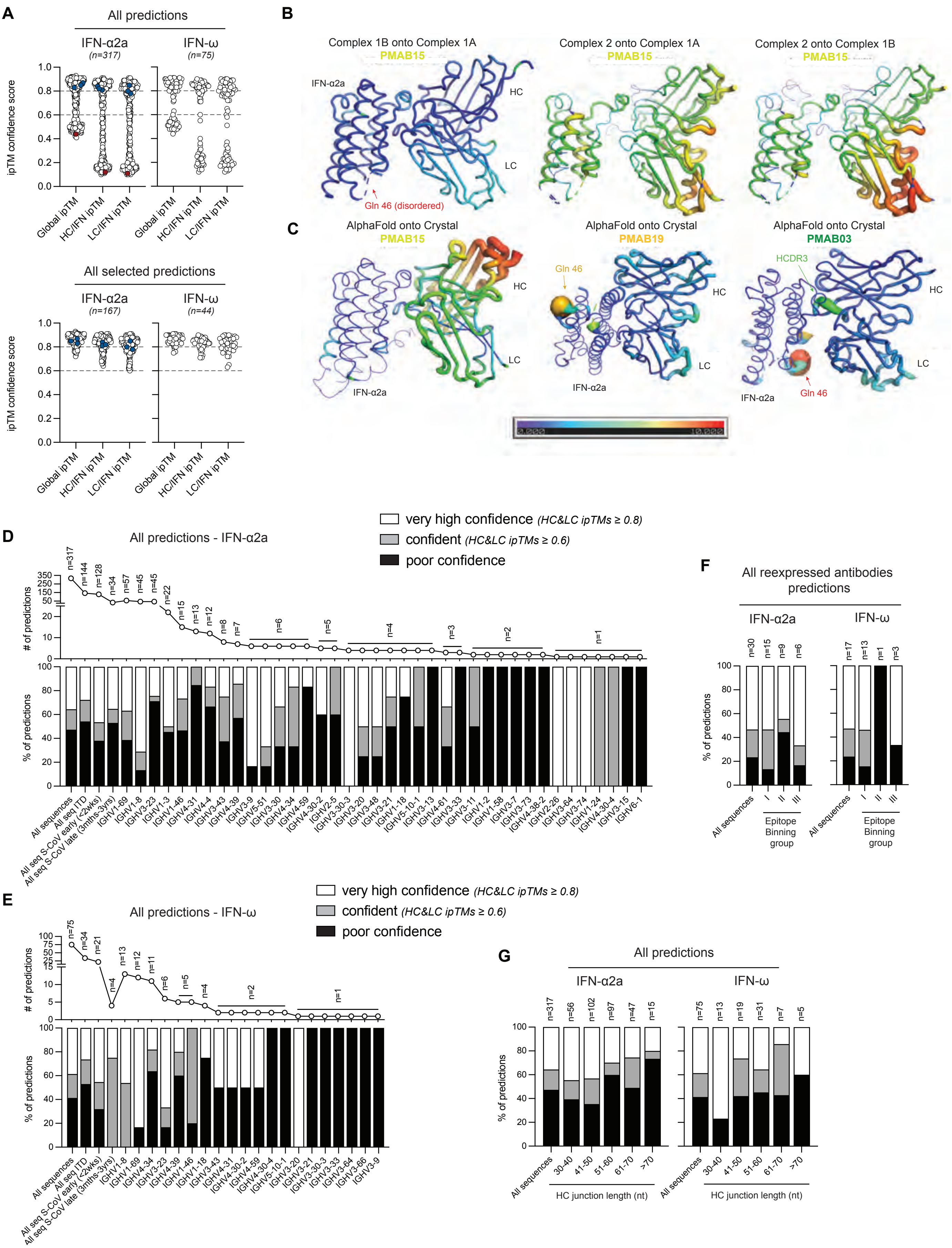

Supplement: Supplementary file 5 [file NIHMS2205108-supplement-Figure_S5.pdf]
