# Supplementary material for: Affinity-matured B cell responses neutralizing type-I interferons underlie severe viral infections
Source: Cell. Author manuscript; Available in PMC 2026 Oct 6. (PMC13639993; doi:10.1016/j.cell.2026.04.013)
Supplement: Supplementary file 8 [file NIHMS2205108-supplement-Table_S4.docx]

**Table S4: RMSDs upon superposition of the three independent X-ray structures of the PMAB15/IFNa2 complex from crystals 1 and 2**

|  | Global  Cmpx1 on Cmpx2^(3)^ | IFN -Xrays 2 aligned on Xray1^(4)^ | PMAB15  X-rays 2^(5)^ | IFN Xrays 3 on Xray1^(6)^ | PMAB15  X-rays 3^(7)^ | IFN Xrays 3 on Xray2^(8)^ | PMAB15  X-rays 3^(9)^ |
| --- | --- | --- | --- | --- | --- | --- | --- |
| RMSD Align^(1)^ (Å):  Number of atoms  RMS_CUR^(2)^ all atoms (Å) Number of atoms  RMS_CUR Ca atoms(Å)  Number of atoms | 0.513 Å  2432  0.855 Å  2784  0.746 Å  359 | 0.157  871  0.486  985  0.185  120 | 1.630  1769  1.561  233 | 0.337  810  0.597  125 | 5.243  232 | 0.395  815  0.619  125 | 5.641  232 |
|  | Global alignment ^(10)^ | |  | | Alignment of Mabs only^(11)^ | | |
| PMAB15 scFv X-rays 1 | Cmpx3 on Cmpx1 | Cmpx3 on Cmpx2 |  |  | scFv3 on scFv1 | scFv3 on scFv2 | scFv2 on scFv1 |
| RMSD Align (Å)  Number of atoms  RMS_CUR all atoms Number of atoms  RMS_CUR Ca atoms(Å)  Number of atoms | 0.905 2387  1.449  2763  1,178  353 | 0.616 Å 2045  1.638 Å 2754  1.460 Å  353 |  |  | 0.213 1491  0.370  231 | 0.224 1471  0.445  231 | 0.205 1503  0.653 1769  0.482  233 |

(1) Program Align within Pymol (The PyMOL Molecular Graphics System, Version 1.2r3pre, Schrödinger, LLC) performs a sequence alignment followed by a structural superposition. It finds the best superposition between two molecules and refines it by eliminating atoms above a certain RMSD cutoff (default value: 2Å)
(2) RMS_CUR, in PYMOL, computes the global RMSD between two molecules without trying to superpose them. It is computed here after Align to inform on the RMSD between all atoms and in particular, of the C𝛼 carbon atoms
(3) Global alignment between the two IFN2a/PMAB15 complex present in the aysmmetric unit of crystal 1 (table SX).
(4) One of the two complexes of the asymmetric unit of crystal 1 (cmpx1) is aligned on the IFN moiety of the second complex (cmpx2)
(5) The RMSDs listed in this column measure how well the two PMAB15 scFvs superpose after alignment of the antigen (IFN2a), after the alignment indicated in ^(4)^ (previous column)
(6) Alignment of the IFN moiety of crystal 2 (IFN2a in ternary complex with the scFvs PMAB15 and PMAB14) on the IFN moiety of cmpx1(crystal 1).
(7) The PMAB15 scFv from crystal superposes significantly less well on its counterpart of cmpx1.
(8) Same as in ^(6)^, but superposing the IFN2a moiety of crystal 2 on that of cmpx2
(9) Same as in ^(7)^ but corresponding to the second scFv of crystal 1, after the alignement indicated in ^(8)^
(10) Cmpx3 is the complex of IFN2a and PMB15 scFv of crystal 2, leaving out the PMB14 scFv
(11) Alignment on the scFv indicates that PMAB15 scFv present in the ternary complex of crystal 2 aligns to either scFv of crystal 1 with a very low RMSD, in the same reange as aligning the two scFvs of crystal 1.
