# Supplementary material for: Affinity-matured B cell responses neutralizing type-I interferons underlie severe viral infections
Source: Cell. Author manuscript; Available in PMC 2026 Oct 6. (PMC13639993; doi:10.1016/j.cell.2026.04.013)
Supplement: Supplementary file 9 [file NIHMS2205108-supplement-Table_S5.docx]

**Table S5**. **RMSD statistics upon alignment of the top 5 AF3 predictions for the IFNa2/PMAB15 complex on the complexes of crystals 1 and 2**

| Alignment on: | IFNa2 -Xrays1 ^(1)^ | IFNa2 -Xrays2 ^(1)^ |
| --- | --- | --- |
| RMSD Align^(3)^ (Å),  Number of atoms:  RMS_CUR^(4)^ all atoms (Å) Number of atoms  RMS_CUR ^(4)^ Ca at. (Å)  Number of Ca atoms | AF3 predictions 0 / 1 / 2 / 3 / 4 ^(2)^  0.416 / 0.411 / 0.407 / 0.403 / 0.404  793 / 794 / 777 / 789 / 810  1.007 / 1.054 / 1.070 / 1.024 / 1.009  1004 / 1004 / 1004 / 1004 / 1004  0.767 / 0.740 / 0.802 / 0.765 / 0.692  125 / 125 / 125 / 125 / 125 | 0.445 / 0.431 / 0.443 / 0.433 / 0.422 829 / 821 / 813 / 824 / 844  0.897 / 0.956 / 0.969 / 0.915 / 0.894 1004 / 1004 / 1004 / 1004 / 1004  0.696 / 0.673 / 0.729 / 0.694 / 0.616  125 / 125 / 125 / 125 / 125 |
| Complex ^(5)^ |  |  |
| RMS_CUR^(4)^ all atoms (Å) Number of atoms:  RMS_CUR^(4)^ Ca atoms(Å)  Number of Ca atoms | 3.769 / 3.615 / 3.670 / 3.755 / 3.784 2773 / 2773 / 2773 / 2773 / 2773  3.769 / 3.598 / 3.670 / 3.762 / 3.750  358 / 358 / 358 / 358 / 358 | 3.028 / 2.809 / 2.885 / 3.027 / 2.829 2773 / 2773 / 2773 / 2773 / 2773  2.999 / 2.757 / 2.832 / 3.006 / 2.766  358 / 358 / 358 / 358 / 358 |
| scFv ^(6)^ |  |  |
| RMS_CUR^(4)^ all atoms (Å) Number of atoms  RMS_CUR^(4)^ Ca atoms(Å)  Number of Ca atoms | 4.658 / 4.455 / 4.523 / 4.638 / 4.676 1769 / 1769 / 1769 / 1769 / 1769  4.637 / 4.427 / 4.495 / 4.629 / 4.621  233 / 233 / 233 / 233 / 233 | 3.730 / 3.442 / 3.537 / 3.727 / 3.478 1745 / 1745 / 1745 / 1745 / 1745  3.683 / 3.382 / 3.470 / 3.691 / 3.398  233 / 233 / 233 / 233 / 233 |

| Alignment on: | X-ray complex 1 ^(7)^ | X-ray complex 2 ^(7)^ |
| --- | --- | --- |
| RMSD Align^(3)^ (Å):  Number of atoms:    RMS_CUR^(4)^ all atoms (Å) Number of atoms:    RMS_CUR^(4)^ Ca atoms(Å)  Number of atoms | 2.120 / 2.018 / 2.003 / 2.107 / 2.068  2691/ 2676/ 2659 / 2705 / 2662    2.327 / 2.243 / 2.261/ 2.296/ 2.322  2773 / 2773 / 2773 / 2773 / 2773    2.247 / 2.158 / 2.261 / 2.220 / 2.244  358 / 358 / 358 / 358 / 358 | 1.747/ 1.645 / 1.658 / 1.726 / 1.702  2726/ 2710/ 2711/ 2723/ 2716    1.886/ 1.806 / 1.827 / 1.864 / 1.850  2773 / 2773 / 2773 / 2773 / 2773    1.769 / 1.672 / 1.679 / 1.752 / 0. 1.731  358 / 358 / 358 / 358 / 358 |
| IFNa2^(8)^ |  |  |
| RMS_CUR^(4)^ all atoms (Å) Number of atoms:    RMS_CUR^(4)^ Ca atoms(Å)  Number of Ca atoms | 2.687 / 2.243 / 2.261/ 2.296 / 2.322  1004 / 1004 / 1004 / 1004 / 1004    2.670 / 2.601/ 2.682 / 2.582 / 2.785  125 / 125 / 125 / 125 / 125 | 1.930 / 1.847 / 1.859 / 1.888 / 1.882  1004 / 1004 / 1004 / 1004 / 1004    1.887 / 1.778 / 1.795 / 1.835 / 1.848  125 / 125 / 125 / 125 / 125 |
| scFv ^(6)^ |  |  |
| RMS_CUR^(4)^ all atoms (Å)  Number of atoms  RMS_CUR^(4)^ Ca atoms(Å) Number of Ca atoms : | 2.095 / 1.994 / 1.970 / 2.098 / 2.020  1769 / 1769 / 1769 / 1769 / 1769  1.983 / 1.877 / 1.835 / 1.999 / 1.891  233 / 233 / 233 / 233 / 233 | 1.860 / 1.782 / 1.808 / 1.851 / 1.831  1769 / 1769 / 1769 / 1769 / 1769  1.703 / 1.612 / 1.614 / 1.707 / 1.665  233 / 233 / 233 / 233 / 233 |

| Alignment on: | IFNa2 -Xrays3^(1)^ | X-ray complex 3 ^(7)^ |
| --- | --- | --- |
| PMAB15 pred. 0/1/2/3/4 RMSD Align^(2)^ (Å), Number of atoms:  RMS_CUR^(4)^ all atoms (Å) Number of atoms  RMS_CUR^(4)^ Ca atoms (Å)  Number of Ca atoms | 0.599 / 0.580 / 0.607 / 0.575 / 0.537  862 / 861 / 836 / 861 / 849  1.287 / 1.279 / 1.410 / 1.282 / 1.280  1106 / 1106 / 1106 / 1106 / 1106  1.090 / 1.041 / 1.166 / 1.056 / 1.020  136 / 136 / 136 / 136 / 136 | 1.326 / 1.211 / 1.249 / 1.400 / 1.226  2302 / 2261 / 2283 / 2360 / 2234  2.706 / 2.672 / 2.673 / 2.564 / 2.895 2886 / 2886 / 2886 / 2886 / 2886  2.598 / 2.562 / 2.577 / 2.461 / 2.789  366 / 366 / 366 / 366 / 366 |
| Complex ^(3)^ | | IFNa2^(8)^ |
| RMS_CUR^(4)^ all atoms (Å) Number of atoms:  RMS_CUR^(4)^ Ca atoms(Å)  Number of Ca atoms | 4.215 / 4.273 / 4.093 / 4.046 / 4.751  2866 / 2866 / 2866 / 2866 / 2866  4.178 / 4.227 / 4.037 / 4.004/ 4.707  366 / 366 / 366 / 366 / 366 | 4.135 / 4.103 / 4.089 / 3.868 / 4.477 1106 / 1106 / 1106 / 1106 / 1106  4.131 / 4.099 / 4.113 / 3.864 / 4.483  136 / 136 / 136 / 136 / 136 |
| scFv ^(4)^ |  |  |
| RMS_CUR^(4)^ all atoms (Å) Number of atoms  RMS_CUR^(4)^ Ca atoms(Å)  Number of Ca atoms | 5.281 / 5.358 / 5.102 / 5.061 / 5.978 1760 / 1760 / 1760 / 1760 / 1760  5.204 / 5.271 / 5.013 / 4.985 / 5.886  230 / 230 / 230 / 230 / 230 | 1.087 / 1.025 / 1.062 / 1.144 / 1.026 1760 / 1760 / 1760 / 1760 / 1760  0.808 / 0.713 / 0.755 / 0.900/ 0.704  230 / 230 / 230 / 230 / 230 |

(1) IFNa1 and IFNa2 refer to the 2 independent molecules of the complexes of crystal 1, and IFNa3 to crystal 2, in which it is in ternary complex IFNA2/PMAB15/PMAB14
(2) These are the five predictions with highest confidence of for the IFNa2/PMAB15 complexes
(3) Program Align within Pymol (The PyMOL Molecular Graphics System, Version 1.2r3pre, Schrödinger, LLC) performs a sequence alignment followed by a structural superposition. It finds the best superposition between two molecules and refines it by eliminating atoms above a certain RMSD cutoff (default value: 2Å)
(4) RMS_CUR, in Pymol, computes the global RMSD between two molecules without trying to superpose them. It is computed here after Align to inform on the RMSD between all atoms and in particular, of the C𝛼 carbon atoms
(5) RMSD values of the IFN2a/PMAB15 complex after alignment only on the IFN2a moiety.
(6) RMSD values considering only atoms of the PMAB15 scFv after alignment on the IFN2a moiety of the complex.
(7) RMSDs after global alignment between the independent IFN2a/PMAB15 complexes onto each other (complexes 1 and 2 in crystal 1 and complex 3 in crystal 2)
(8) RMSDs of the IFN2a atoms after global alignment of the PMAB15/IFNa2 complex
