# Supplementary material for: Affinity-matured B cell responses neutralizing type-I interferons underlie severe viral infections
Source: Cell. Author manuscript; Available in PMC 2026 Oct 6. (PMC13639993; doi:10.1016/j.cell.2026.04.013)
Supplement: Supplementary file 10 [file NIHMS2205108-supplement-Table_S6.docx]

**Table S6**. **RMSDs upon superposition of the quaternary complex of crystal 3 with the 5 top AF3 prediction for the respective complexes**

|  | A - Alignment on IFNa2 | | |
| --- | --- | --- | --- |
|  | **PMAB19** pred. 0/1/2/3/4 | **PMAB14** pred. 0/1/2/3/4 | **PMAB03** pred. 0/1/2/3/4 |
| RMSD Align (Å), | 0.478 / 0.526 / 0.429 / 0. 526 / 0. 509 | 0.466 / 0.413 / 0.397 / 0.427 / 0.431 | 0.446 / 0.504 / 0.330 / 0.496 / 0.478 |
| # of atoms | 925 / 933 / 916 / 947 / 937 | 910 / 881 / 887 / 906 / 937 | 925 / 943 / 882 / 909 / 912 |
| RMSD between corresponding IFN atoms in the X-ray and in the 5 predictions after alignment on the IFN | | | |
| RMS_CUR all atoms (Å) | 2.067 / 2.124 / 1.932 / 2.059 / 1.926 | 2.063 / 2.103 / 1.993 / 2.048 / 1.941 | 1.115/ 1.846/ 1.998 / 2.115 / 1.980 |
| Number of atoms | 1172 / 1172 / 1172 / 1172 / 1172 | 1183 / 1183 / 1183 / 1183 / 1183 | 1172 / 1172 / 1172 / 1172 / 1172 |
| RMS_CUR C𝛼 atoms (Å) | 1.294 / 1.371 / 1.120 / 1.248 / 1.328 | 1.275 / 1.397 / 1.288 / 1.350 / 1.350 | 0.621 / 1.086 / 1.215 / 1.356 / 1.223 |
| Number of atoms | 143 / 143 / 143 / 143 / 143 | 144 / 144 / 144 / 144 / 144 | 143 / 143 / 143 / 143 / 143 |
| RMSD between corresponding scFv atoms in the X-ray and in the 5 predictions after alignment on the IFN | | | |
| RMS_CUR all atoms (Å) | 1.613 / 1.758 / 1.750 / 1.841 / 1.859 | 38.7 / 45.7 / 45.8 / 48.5 / 55.7 | 2.266 / 1.956 / 2.347 / 2.664 / 2.531 |
| Number of atoms | 1684 / 1684 / 1684 / 1684 / 1684 | 1709 / 1709 / 1709 / 1709 / 1709 | 1715 / 1715 / 1715 / 1715 / 1715 |
| RMS_CUR C𝛼 atoms(Å) | 1.505/ 1.687 / 1.674 / 1.769 / 1.784 | 38.8 / 45.8 / 45.9 / 48.6 / 55.7 | 2.208 / 1.898 / 2.303 / 2.638 / 2.485 |
| Number of C𝛼 atoms | 220 / 220 / 220 / 220 / 220 | 226 / 226 / 226 / 226 / 226 | 226 / 226 / 226 / 226 / 226 |
| RMSD between corresponding atoms in each scFv/IFN complex in the X-ray and in the 5 predictions after alignment on the IFN | | | |
| RMS_CUR all atoms (Å) | 1.813 / 1.916 / 1.827 / 1.933 / 1.887 | 29.8 / 35.1 / 35.2 / 37.3 / 42.8 | 1.885 / 1.912 / 2.212/ 2.456 / 2.323 |
| Number of atoms | 2856 / 2856 / 2856 / 2856 / 2856 | 2892 / 2892 / 2892 / 2892/2892 | 2887 / 2887 / 2887 / 2887 / 2887 |
| RMS_CUR C𝛼 atoms(Å) | 1.425 / 1.570 / 1.481/ 1.584 / 1.620 | 30.35 / 35.8 / 35.9 / 38.0 / 43.6 | 1.771 / 1.632/ 1.955 / 2.230 / 2.088 |
| Number of C𝛼 atoms | 363 / 363 / 363 / 363 / 363 | 370 / 370 / 370 / 370 / 370 | 369 / 369 / 369 / 369 / 369 |

|  | B - Overall Alignment | | | |
| --- | --- | --- | --- | --- |
|  | **PMAB19** pred. 0/1/2/3/4 | **PMAB14** pred. 0/1/2/3/4 | | **PMAB03** pred. 0/1/2/3/4 |
| RMSD Align (Å), # of atoms | 0.591 / 0.640 / 0.588 / 0. 638 / 0. 634  2426 / 2443 / 2468 / 2443 / 2474 | 8.185 / 6.140 / 7.751 / 7.772 / 3.637  2382 2092 / 2215 / 2127 / 1929 | | 0.446 / 0.504 / 0.330 / 0.496 / 0.478  925 / 943 / 882 / 909 / 912 |
| IFN moiety only: |  |  | |  |
| RMS_CUR all atoms (Å) | 2.183 / 2.278 / 1.989 / 2.168 / 2.056 | 20.9 / 27.2 / 27.1 / 29.8 / 24.9 | | 1.115/ 1.846/ 1.998 / 2.115 / 1.980 |
| Number of atoms | 1172 / 1172 / 1172 / 1172 / 1172 | 1183 / 1183 / 1183 / 1183 / 1183 | | 1172 / 1172 / 1172 / 1172 / 1172 |
| RMS_CUR Ca atoms (Å) | 1.435 / 1.558 / 1.182 / 1.387 / 1.468 | 20.9 / 27.4 / 27.2 / 30.0 / 24.5 | | 0.621 / 1.086 / 1.215 / 1.356 / 1.223 |
| Number of atoms | 143 / 143 / 143 / 143 / 143 | 144 / 144 / 144 / 144 / 144 | | 143 / 143 / 143 / 143 / 143 |
| RMSD between the IFN moiety of the 5 predictions (in Å for 144 Ca) after global alignment on the corresponding X-ray structure: | | | | |
| 0:1, 0:2, 0:3, 0:4, 1:2, | 0.37 / 1.1 / 0.71 / 0.53 / 1.10 | 12.2 / 11.1 / 17.1 / 27.6 | / 2.0 | 0.91 / 1.16 / 1.23 / 1.10 / 0.69 |
| 1:3, 1:4, 2:3, 2:4, 3:4 | 0.71 / 0.43 / 0.67 / 1.15 / 0.79 | 6.3 / 29.8 / 7.7 / 27.8 | / 2.2 | 0.72 / 0.70 / 0.37 / 0.36 / 0.38 |
| scFv moiety only: |  |  | |  |
| RMS_CUR all atoms (Å) | 0.899 / 0.826 / 0.814 / 0.836 / 0.825 | 5.465 / 3.350 / 4.550 / 4.551 / 2.348 | | 2.266 / 1.956 / 2.347 / 2.664 / 2.531 |
| Number of atoms | 1684 / 1684 / 1684 / 1684 / 1684 | 1709 / 1709 / 1709 / 1709 / 1709 | | 1715 / 1715 / 1715 / 1715 / 1715 |
| RMS_CUR Ca atoms(Å) | 0.594/ 0.600 / 0.580 / 0.598 / 0.596 | 5.248 / 3.069 / 4.321 / 4.224 / 1.963 | | 2.208 / 1.898 / 2.303 / 2.638 / 2.485 |
| Number of Ca atoms | 220 / 220 / 220 / 220 / 220 | 226 / 226 / 226 / 226 / 226 | | 226 / 226 / 226 / 226 / 226 |
| RMSD between the scFv moiety of the 5 predictions (in Å for 226 Ca) after global alignment on the corresponding X-ray structure: | | | | |
| 0:1/ 0:2/0:3/ 0:4/ 1:2 | 0.17 / 0.16 / 0.20 / 0.20 / 0.13 | 2.5 / 1.6 / 2.2 / 4.2 / 1.4 | | 0.67 / 0.69 / 0.52 / 0.61 / 0.61 |
| 1:3/ 1:4/ 2:3/ 2:4/3:4 | 0.11 / 0.11 / 0.14 / 0.15 / 0.17 | 1.5 / 1.9 / 1.0 / 3.0 / 2.9 | | 1.13 / 0.80 / 0.99 / 0.59 / 0.76 |
| scFv/IFN complexes: | PMAB19/IFNa2 X-ray complex | PMAB14/IFNa2 X-ray complex | | PMAB03/IFNa2 X-rays |
| RMS_CUR all atoms (Å) | 1.559 / 1.591 / 1.419 / 1.530 / 1.461 | 14.0 / 17.6 / 17.7 / 19.4 / 16.1 | | 1.885 / 1.912 / 2.212/ 2.456 / 2.323 2887 / |
| Number of atoms | 2856 / 2856 / 2856 / 2856 / 2856 | 2893 / 2893 / 2893 / 2893 / 2893 | | 2887 / 2887 / 2887 / 2887 |
| RMS_CUR Ca atoms(Å) | 1.012 / 1.084 / 0.868 / 0.987 / 1.032 | 13.7 / 17.3 / 17.3 / 19.0 / 15.4 | | 1.771 / 1.632/ 1.955 / 2.230 / 2.088 |
| Number of Ca atoms | 363 / 363 / 363 / 363 / 363 | 370 / 370 / 370 / 370 / 370 | | 369 / 369 / 369 / 369 / 369 |
