# Supplementary material for: Affinity-matured B cell responses neutralizing type-I interferons underlie severe viral infections
Source: Cell. Author manuscript; Available in PMC 2026 Oct 6. (PMC13639993; doi:10.1016/j.cell.2026.04.013)
Supplement: Supplementary file 11 [file NIHMS2205108-supplement-Table_S7.docx]

**Table S7. RMSDs upon alignment against each other the 5 top AF3 predictions of of the complexes that were confirmed by the X-ray structures.**

| **pmab19_1^(1)^**  RMSD Align:  Number of atoms  RMS_CUR all atoms (Å)  Number of atoms  RMS_CUR Ca atoms(Å)  Number of atoms | pmab19_0 | pmab19_1 | pmab19_2 | pmab19_3 |
| --- | --- | --- | --- | --- |
|  | 0.136 Å  2516  0.597 Å  3069  0.324 Å  389 |  |  |  |
| **pmab19_2**  RMSD Align:  Number of atoms  RMS_CUR all atoms (Å)  Number of atoms  RMS_CUR Ca atoms(Å)  Number of atoms |  | | | |
|  | 0.137 Å  2448  3.020 Å  3069  2.596Å  389 | 0.163 Å  2509  2.961 Å  3069  2.571 Å  389 |  |  |
| **pmab19_3**  RMSD Align:  Number of atoms:  RMS_CUR all atoms:  Number of atoms:  RMS_CUR Ca atoms:  Number of Ca atoms: |  | | | |
|  | 0.167 Å  2466  0.984 Å  3069  0.592 Å  389 | 0.111 Å  2506  0.837 Å  3069  0.549 Å  389 | 0.164 Å  2540  2.702 Å  3069  2.311Å  389 |  |
| **pmab19_4**  RMSD Align:  Number of atoms  RMS_CUR all atoms:  Number of atoms:  RMS_CUR Ca atoms:  Number of Ca atoms: |  | | | |
|  | 0.167 Å  2606  0.785 Å  3069  0.393 Å  389 | 0.140 Å  2596  0.635 Å  3069  0.294Å  389 | 0.168 Å  2649  3.050 Å  3069  2.634 Å  389 | 0.175 Å  2582  1.050 Å  3069  0.626Å  389 |

| **sel19_1^(2)^**  RMSD Align:  Number of atoms  RMS_CUR all atoms (Å)  Number of atoms  RMS_CUR Ca atoms(Å)  Number of atoms | sel19_0 | sel19_1 | sel19_2 | sel19_3 |
| --- | --- | --- | --- | --- |
|  | 0.133 Å  2476  0.577 Å  2893  0.288 Å  366 |  |  |  |
| **sel19_2**  RMSD Align:  Number of atoms  RMS_CUR all atoms (Å)  Number of atoms  RMS_CUR Ca atoms(Å)  Number of atoms |  |  |  |  |
|  | 0.131 Å  2386  0.991 Å  2893  0.567 Å  366 | 0.155 Å  2419  0.910 Å  2893  0.583 Å  366 |  |  |
| **sel19_3**  RMSD Align:  Number of atoms:  RMS_CUR all atoms:  Number of atoms:  RMS_CUR Ca atoms:  Number of Ca atoms: |  |  |  |  |
|  | 0.163 Å  2420  0.930 Å  2893  0.487 Å  366 | 0.108 Å  2454  0.784 Å  2893  0.455 Å  366 | 0.157 Å  2463  0.542 Å  2893  0.248 Å  366 |  |
| **sel19_4**  RMSD Align:  Number of atoms  RMS_CUR all atoms:  Number of atoms:  RMS_CUR Ca atoms:  Number of Ca atoms: |  |  |  |  |
|  | 0.160 Å  2481  0.781 Å  2893  0.377 Å  366 | 0.136 Å  2536  0.638 Å  2893  0.284  366 | 0.167 Å  2616  1.071 Å  2893  0.622 Å  366 | 0.172 Å  2536  0.998 Å  2893  0.519  366 |

| **pmab03_1^(1)^**  RMSD Align:  Number of atoms  RMS_CUR all atoms (Å)  Number of atoms  RMS_CUR Ca atoms(Å)  Number of atoms | pmab03_0 | pmab03_1 | pmab03_2 | pmab03_3 |
| --- | --- | --- | --- | --- |
|  | 0.232Å  2648  1.058 Å  3076  0.661 Å  392 |  |  |  |
| **pmab03_2**  RMSD Align:  Number of atoms  RMS_CUR all atoms (Å)  Number of atoms  RMS_CUR Ca atoms(Å)  Number of atoms |  |  |  |  |
|  | 0.211Å  2451  2.588 Å  3076  2.596Å  392 | 0.303 Å  2694  2.437 Å  3076  2.103 Å  392 |  |  |
| **pmab03_3**  RMSD Align:  Number of atoms:  RMS_CUR all atoms:  Number of atoms:  RMS_CUR Ca atoms:  Number of Ca atoms: |  |  |  |  |
|  | 0.150 Å  2396  2.623 Å  3076  2.160 Å  392 | 0.303 Å  2589  2.503 Å  3076  2.138 Å  392 | 0.286 Å  2490  0.647 Å  3076  0.505 Å  392 |  |
| **pmab03_4**  RMSD Align:  Number of atoms  RMS_CUR all atoms:  Number of atoms:  RMS_CUR Ca atoms:  Number of Ca atoms: |  |  |  |  |
|  | 0.243 Å  2561  2.579 Å  3076  2.148 Å  392 | 0.300 Å  2653  2.479 Å  3076  2.140 Å  392 | 0.254 Å  2629  0.570 Å  3076  0.437 Å  392 | 0.169 Å  2388  0.564 Å  3076  0.428 Å  392 |

| **sel03_1^(2)^**  RMSD Align:  Number of atoms  RMS_CUR all atoms (Å)  Number of atoms  RMS_CUR Ca atoms(Å)  Number of atoms | sel03_0 | sel03_1 | sel03_2 | sel03_3 |
| --- | --- | --- | --- | --- |
|  | 0.225 Å  2558  1.046 Å  2917  0.638 Å  371 |  |  |  |
| **sel03_2**  RMSD Align:  Number of atoms  RMS_CUR all atoms (Å)  Number of atoms  RMS_CUR Ca atoms(Å)  Number of atoms |  |  |  |  |
|  | 0.196 Å  2348  1.164 Å  2917  0.747 Å  371 | 0.294 Å  2622  0.598 Å  2917  0.396 Å  371 |  |  |
| **sel03_3**  RMSD Align:  Number of atoms:  RMS_CUR all atoms:  Number of atoms:  RMS_CUR Ca atoms:  Number of Ca atoms: |  |  |  |  |
|  | 0.146 Å  2360  1.135 Å  2917  0.677 Å  371 | 0.280 Å  2473  0.642 Å  2917  0.415 Å  371 | 0.257 Å  2355  0.614 Å  3076  0.477 Å  371 |  |
| **sel03_4**  RMSD Align:  Number of atoms  RMS_CUR all atoms:  Number of atoms:  RMS_CUR Ca atoms:  Number of Ca atoms: |  |  |  |  |
|  | 0.237 Å  2521  1.069 Å  2917  0.629 Å  371 | 0.294 Å  2917  0.602 Å  2917  0.390 Å  371 | 0.242 Å  2516  0.559 Å  2917  0.426 Å  371 | 0.159 Å  2315  0.510 Å  2917  0.361 Å  371 |

| **pmab15_1^(1)^**  RMSD Align:  Number of atoms  RMS_CUR all atoms (Å)  Number of atoms  RMS_CUR Ca atoms(Å)  Number of atoms | pmab15_0 | pmab15_1 | pmab15_2 | pmab15_3 |
| --- | --- | --- | --- | --- |
|  | 0.162 Å  2722  1.090 Å  3131  0.813 Å  398 |  |  |  |
| **pmab15_2**  RMSD Align:  Number of atoms  RMS_CUR all atoms (Å)  Number of atoms  RMS_CUR Ca atoms(Å)  Number of atoms |  |  |  |  |
|  | 0.144 Å  2602  1.233 Å  3131  0.914 Å  398 | 0.121 Å  2510  0.567 Å  3131  0.390 Å  398 |  |  |
| **pmab15_3**  RMSD Align:  Number of atoms:  RMS_CUR all atoms:  Number of atoms:  RMS_CUR Ca atoms:  Number of Ca atoms: |  |  |  |  |
|  | 0.163 Å  2705  0.923 Å  3131  0.750 Å  398 | 0.189 Å  2589  0.700 Å  3131  0.355 Å  398 | 0.176 Å  2646  0.866 Å  3131  0.515 Å  398 |  |
| **pmab15_4**  RMSD Align:  Number of atoms  RMS_CUR all atoms:  Number of atoms:  RMS_CUR Ca atoms:  Number of Ca atoms: |  |  |  |  |
|  | 0.182 Å  2361  1.388 Å  3131  0.965 Å  398 | 0.176 Å  2653  1.194 Å  3131  0.642 Å  398 | 0.179 Å  2405  1.360 Å  3131  0.786 Å  398 | 0.259 Å  2556  1.103 Å  3131  0.636 Å  398 |

| **sel15_1^(2)^**  RMSD Align:  Number of atoms  RMS_CUR all atoms (Å)  Number of atoms  RMS_CUR Ca atoms(Å)  Number of atoms | sel15_0 | sel15_1 | sel15_2 | sel15_3 |
| --- | --- | --- | --- | --- |
|  | 0.159Å  2606  0.336 Å  2773  0.169Å  358 |  |  |  |
| **sel15_2**  RMSD Align:  Number of atoms  RMS_CUR all atoms (Å)  Number of atoms  RMS_CUR Ca atoms(Å)  Number of atoms |  |  |  |  |
|  | 0.139 Å  2520  0.409 Å  2773  0.219 Å  358 | 0.116 Å  2423  0.359 Å  2773  0.203 Å  358 |  |  |
| **sel15_3**  RMSD Align:  Number of atoms:  RMS_CUR all atoms:  Number of atoms:  RMS_CUR Ca atoms:  Number of Ca atoms: |  |  |  |  |
|  | 0.157 Å  2594  0.303 Å  2773  0.165 Å  358 | 0.182 Å  2643  0.336 Å  2773  0.183 Å  358 | 0.172 Å  2552  0.406 Å  2773  0.244 Å  358 |  |
| **sel15_4**  RMSD Align:  Number of atoms  RMS_CUR all atoms:  Number of atoms:  RMS_CUR Ca atoms:  Number of Ca atoms: |  |  |  |  |
|  | 0.131 Å  2051  0.435 Å  2773  0.312 Å  358 | 0.140 Å  2211  0.417 Å  2773  0.261 Å  358 | 0.139 Å  2122  0.487 Å  2773  0.314 Å  358 | 0.191 Å  2202  0.484 Å  2773  0.378 Å  358 |

^(1)^These comparisons use the entire amino acid sequence of IFNa2 and the scFv to compute the listed RMSDs, including disordered/mobile regions not seen in the X-ray structures

^(2)^ Disordered/mobile regions not seen in the X-ray structures of IFNa2 and the scFvs were removed to compute the listed RMSDs
